# Endophyte-mediated enhancement of salt resistance in *Arachis hypogaea* L. by regulation of osmotic stress and plant defense-related genes

**DOI:** 10.3389/fmicb.2024.1383545

**Published:** 2024-05-23

**Authors:** Qihua Liang, Dedong Tan, Haohai Chen, Xiaoli Guo, Muhammad Afzal, Xiaolin Wang, Zhiyuan Tan, Guixiang Peng

**Affiliations:** ^1^College of Natural Resources and Environment, South China Agricultural University, Guangzhou, China; ^2^University of South China, Hengyang, China; ^3^College of Agriculture, South China Agricultural University, Guangzhou, China

**Keywords:** soil salinization, endophytes, *Arachis hypogaea* L., transcriptome response, immune effects, antioxidant enzymes, secondary metabolism

## Abstract

**Introduction:**

Soil salinization poses a significant environmental challenge affecting plant growth and agricultural sustainability. This study explores the potential of salt-tolerant endophytes to mitigate the adverse effects of soil salinization, emphasizing their impact on the development and resistance of *Arachis hypogaea* L. (peanuts).

**Methods:**

The diversity of culturable plant endophytic bacteria associated with *Miscanthus lutarioriparius* was investigated. The study focused on the effects of *Bacillus tequilensis*, *Staphylococcus epidermidis*, and *Bacillus siamensis* on the development and germination of *A. hypogaea* seeds in pots subjected to high NaCl concentrations (200 mM L^−1^).

**Results:**

Under elevated NaCl concentrations, the inoculation of endophytes significantly (*p* < 0.05) enhanced seedling germination and increased the activities of enzymes such as Superoxide dismutase, catalase, and polyphenol oxidase, while reducing malondialdehyde and peroxidase levels. Additionally, endophyte inoculation resulted in increased root surface area, plant height, biomass contents, and leaf surface area of peanuts under NaCl stress. Transcriptome data revealed an augmented defense and resistance response induced by the applied endophyte (*B. tequilensis*, *S. epidermidis*, and *B. siamensis*) strain, including upregulation of abiotic stress related mechanisms such as fat metabolism, hormones, and glycosyl inositol phosphorylceramide (Na^+^ receptor). Na^+^ receptor under salt stress gate Ca^2+^ influx channels in plants. Notably, the synthesis of secondary metabolites, especially genes related to terpene and phenylpropanoid pathways, was highly regulated.

**Conclusion:**

The inoculated endophytes played a possible role in enhancing salt tolerance in peanuts. Future investigations should explore protein–protein interactions between plants and endophytes to unravel the mechanisms underlying endophyte-mediated salt resistance in plants.

## Introduction

Soil salinity poses a significant challenge to agricultural land, hampering plant growth and reducing yields, thus contributing to global food shortages. Currently, approximately 20% of arable land worldwide is affected by salinization (Singh, 2021). The primary challenges faced by crops include the absence of modern agricultural practices, a scarcity of freshwater resources, and the pervasive issue of soil salinity (Choudhary et al., 2023). Without intervention, this issue could affect more than half of the world’s irrigated land by 2050 (Singh, 2021). Addressing this issue is crucial to meet the growing demands for food security.

Salinity and drought have the potential to alter the electron transport chain, photosystems (PS), and enzymes associated with photosynthesis, as well as impact stomatal function and the Calvin cycle. Additionally, salt stress has been shown to impede seed germination, cell signaling, and lipid metabolism in plants (Gupta et al., 2022). Furthermore, salt stress hindered seed germination, cell signaling, and lipid metabolism of the plant (Pan et al., 2023; Ravi et al., 2023; Zhan et al., 2023). Nonetheless, the application of microorganisms has been identified as a potential protective measure, offering a means by which plants can defend against various biotic and abiotic stresses (Khan et al., 2021).

Peanut (*A. hypogaea*) holds significant importance as an oilseed species and stands out as a primary source of edible oil globally (Chen et al., 2023). While peanuts exhibit a moderate resistance to soil salinization compared to other crops, this can still lead to a notable decline in quality (Qin et al., 2021). Consequently, peanuts emerge as an ideal crop for cultivation in saline soils, and a comprehensive understanding of the metabolic adaptation mechanisms to salt tolerance in peanuts is pivotal for the development of eco-friendly and sustainable agricultural practices.

Salt-tolerant peanuts can acclimate to high salinity soils, suggesting that they may engage a series of microorganisms, leading to a reorganization of diverse microbial community structures. In this context, root-associated microorganisms, particularly endophytes (bacteria, archaea, fungi), emerge as a potential microbial group for enhancing salt tolerance in plants (Egamberdieva et al., 2019; Ondrasek et al., 2022). The plant-microbial association represents a mutually beneficial relationship where microbes and plants mutually support each other for nutrients and growth (Kaur and Sharma, 2021).

Salt-tolerant endophytic bacteria serve as potent plant growth-promoting bacteria (PGPB), with a multitude of species displaying the capability to alleviate the detrimental effects of salinity. Noteworthy PGPBs encompass *Arhrobacter*, *Alcaligenes*, *Azospirillum*, *Agrobacterium*, *Bacillus*, *Burkholderia*, *Ochromobacter*, *Enterobacter*, *Klebsiella*, *Microbacterium*, *Pseudomonas*, *Pantoea*, *Streptomyces*, and *Rhizobium*, among others (Sagar et al., 2022). Studies underscore the positive impact of extracellular polysaccharides (EPS) secreted by these bacteria in promoting plant growth under salt stress. For instance, *Bacillus thuringiensis* PM25 enhances nutrient availability in the maize plant rhizosphere by secreting EPS (Ali et al., 2022a). Moreover, the formation of biofilms results in the accumulation of EPS around the roots, exerting a beneficial influence by restricting the accessible Na^+^ content surrounding the plant roots and thus minimizing the deleterious effects of soil salt. In a separate study, strains of *Bacillus mycoides* PM35 demonstrated the ability to enhance the synthesis of photosynthetic pigments, carotenoids, soluble sugars, and protein content, while also boosting the capacity to scavenge free radicals, thereby mitigating salt stress in maize plants (Ali et al., 2022b). Thus, microorganisms capable of producing essential plant nutrients like EPS, iron carriers, and Indole-3-acetic acid (IAA) hold the potential in enhancing vegetative growth and mitigate salt stress.

Transcriptomics analysis, employing high-throughput sequencing, significantly contributes to our comprehension of gene expression levels in plants under salinity stress (Ganie et al., 2021). Moreover, limited reports have delved into the interaction between microorganisms and *A. hypogaea* under salt stress conditions. Experiments have highlighted the positive impact of the plant growth-promoting inter-root bacterium *Brachybacterium saurashtrense* on nitrogen-deficient peanuts under salt stress (Alexander et al., 2022). Transcription factors (TFs) play a crucial role in modulating and regulating the expression of different genes under salinity conditions, demonstrating gene expression association with the promoter region (Arif et al., 2020). Currently, numerous TFs have been identified to play pivotal roles in the growth and development of peanuts, with the *WRKY* family being noteworthy. *WRKY* stands out as one of the largest families of transcriptional regulatory genes in plants, exerting significant influence in abiotic stress response and developmental processes (Wu et al., 2022).

In developing countries, grappling with expanding populations and diminishing arable land, there exists a pressing need for a salt-resistant bacterial agent boasting high yield potential for integration into agricultural production. In the evolving landscape of agricultural practices, the foliar spraying of microorganisms as fertilizers emerges as a potential solution to prevent salt accumulation in plant leaves. This innovative approach holds promise in enhancing crop yield and fostering improved plant growth in salt-stressed conditions. Furthermore, knowledge related to bacterial community colonization help us in selection and inoculation of beneficial bacteria, offering a means to mitigate the adverse impact of salinity on peanut production partially. This strategic undertaking not only lays the groundwork theoretically for elevating peanut production but also bears practical significance. This study aims to: (1) identify key microorganisms influencing plant growth and phytoremediation, (2) examine microbial associations’ impact on plant growth and soil restoration in saline conditions, and (3) understand how microbial communities enhance phytoremediation efficiency. Specifically, we studied the plant’s transcriptome under salt and applied microbes to know difference in plant’s genes expression with applied microbes under salt stress.

## Materials and methods

### Isolation and identification of salt-tolerant endophytic bacteria

Healthy *M. lutarioriparius* specimens were collected in Anqing County, Anhui Province, China (115°45′E, 29°47′N). The *M. lutarioriparius* samples underwent surface sterilization using 70% ethanol for 10 min 3% sodium hypochlorite for 10 min, and were subsequently rinsed eight times with sterile distilled water. A total of 200.0 μL of milled and diluted plant root, stem, and leaf sap was taken and incubated in LB solid medium (containing Peptone 10.0 g L^−1^, NaCl 10.0 g L^−1^, Yeast extract 5.0 g L^−1^, and Agar 20.0 g L^−1^) for 48 h. After 3 days of incubation, emergent colonies were subcultured to obtain pure cultures, which were then subjected to a salt-tolerance test. NaCl treatments (0, 2, 4, 8, 12, 16%) were incorporated into the medium by adding Peptone 10.0 g L^−1^, Yeast extract 5.0 g L^−1^, and 20.0 g L^−1^ Agar, followed by incubation at a constant temperature of 37°C for 48 h.

### Biochemical tests

Following standardized protocols, the isolated strains underwent physiological and biochemical tests, including Voges-Proskauer, indole, urease, gelatin liquefaction, sucrose and citrate utilization, ammonia production, and methyl red, by Bergey’s Manual of Determinative Bacteriology (Breed et al., 1944). The determination of protease activity involved measuring the amount of tyrosine released from the hydrolysis of casein in the reaction mixture (Anson, 1938). The ability of bacteria to produce protease was indicated by the formation of distinct hydrolysis circles in Skimmed milk medium containing Skimmed milk powder (35.0 g L^−1^) and Agar (18.0 g L^−1^).

Bacteria were isolated on media containing carboxymethyl cellulose (CMC) using a modified standard method (Balla et al., 2022). The cellulase activity of the strains was assessed using sodium carboxymethyl cellulose as a substrate. The hydrolysis of cellulose facilitates its conversion to glucose, the primary source of carbohydrates during seed germination. Iron carrier levels in the strains were evaluated using chromium azurite. The phosphate solubilization potential was determined in a medium supplemented with insoluble tricalcium phosphate and quantified following a standard protocol (Sun et al., 2023). Plates were incubated at 28°C for 7 days, and a clear transparent zone for phosphate solubilization around each colony was observed.

A modified method was used for the isolation of amylase-producing strains. Briefly, cultured in a basal medium (pH = 5.5) containing 6.8% (w/v) soluble starch and 2% (w/v) NaCl (Takenaka et al., 2015). Starch produces a blue color when exposed to an iodine solution, so amylase-producing microorganisms treated with iodine produced a colorless area, allowing for the distinction of amylase-producing microorganisms (Ishii et al., 1986). A 20% glycerol were used to preserve the strains in 3 tubes. While one tube was utilized for subsequent experiments and kept at −20°C, the remaining two were stored at −80°C.

### Isolates identification on the basis of 16S *rRNA* gene

Bacterial 16S *rRNA* gene sequencing was conducted at Guangzhou Tianyi Huiyuan Gene Technology Co. Ltd. to molecularly identify isolates. The 16S *rRNA* gene of fresh colonies was amplified using primers 27F (5′-AGA GTT TGA TCC TGG CTC AG-3′) and 1492R (5′-TAC GGY TAC CTT GTT AYG ACT T-3′), subsequently sequenced through the Sanger method at Guangzhou Tianyi Huiyuan Gene Technology Co. Ltd. The obtained sequences were then trimming and aligned with the NCBI library for species annotation classification. Pairwise evolutionary distances between the 16S *rRNA* sequences of the test strains and related bacterial strains were calculated using MEGA software, employing a bootstrap of 1,000 replicates for clustering related taxa.

### Seed germination

The experiments were conducted at the College of Genetic Breeding of Crops, South China Agricultural University, China. The salt-tolerant peanut cultivar Huayu 25 (HY25) served as the experimental material. Surface-sterilized peanut seeds were placed in a growth chamber under controlled environmental conditions: a temperature of 27 ± 1°C (day/night, 0/24 h) and approximately 80% humidity. The seeds were gauze-wrapped and incubated for 3 days to promote germination.

All experiments were performed in triplicate unless otherwise specified. A total of 5 NaCl levels (0 mM L^−1^, 50 mM L^−1^, 100 mM L^−1^, 200 mM L^−1^, 250 mM L^−1^) were employed for the seed germination experiment following protocols from Ju et al. (2021) and Pal et al. (2021). Peanut seedlings were then immersed for 12 h in four different strain combinations (G62 + J1 + J40, G62 + J1 + Y2, J1 + J40 + Y2, G62 + J1 + J40 + Y2) at a concentration of 1 × 10^9^ cfu mL^−1^, while the control group (CK) received LB liquid medium instead of the bacterial solution. After washing the seeds with sterile water, three seeds were transferred to a tissue culture container (height 9.9 cm × diameter 6.7 cm, mouth diameter 5.1 cm). All containers were placed in an incubator at a temperature of 28 ± 1°C (day/night, 14/10 h, and light intensity of 8,000 lx) and maintained at approximately 60 ± 10% humidity for 10 days. After the experiment, measurements were taken for plant height, root length, dry weight (DW), and fresh weight (FW) across all treatments.

### Pots experiment

After careful screening and significantly improving seed germination, we selected a mixture of three bacterial strains, namely TSB (containing *B. tequilensis*, *S. epidermidis*, and *B. siamensis*), where T represents *tequilensis*, S stands for *Staphylococcus*, and B stands for *Bacillus*. This bacterial mixture was utilized for a pot experiment involving peanuts, specifically the HY25 cultivar. Seeds of HY25 were subjected to surface sterilization by immersion in 70% ethanol and 3% sodium hypochlorite for 3 min. Following sterilization, the seeds were thoroughly washed with sterile water to eliminate any residual sodium hypochlorite.

**Figure 1:**
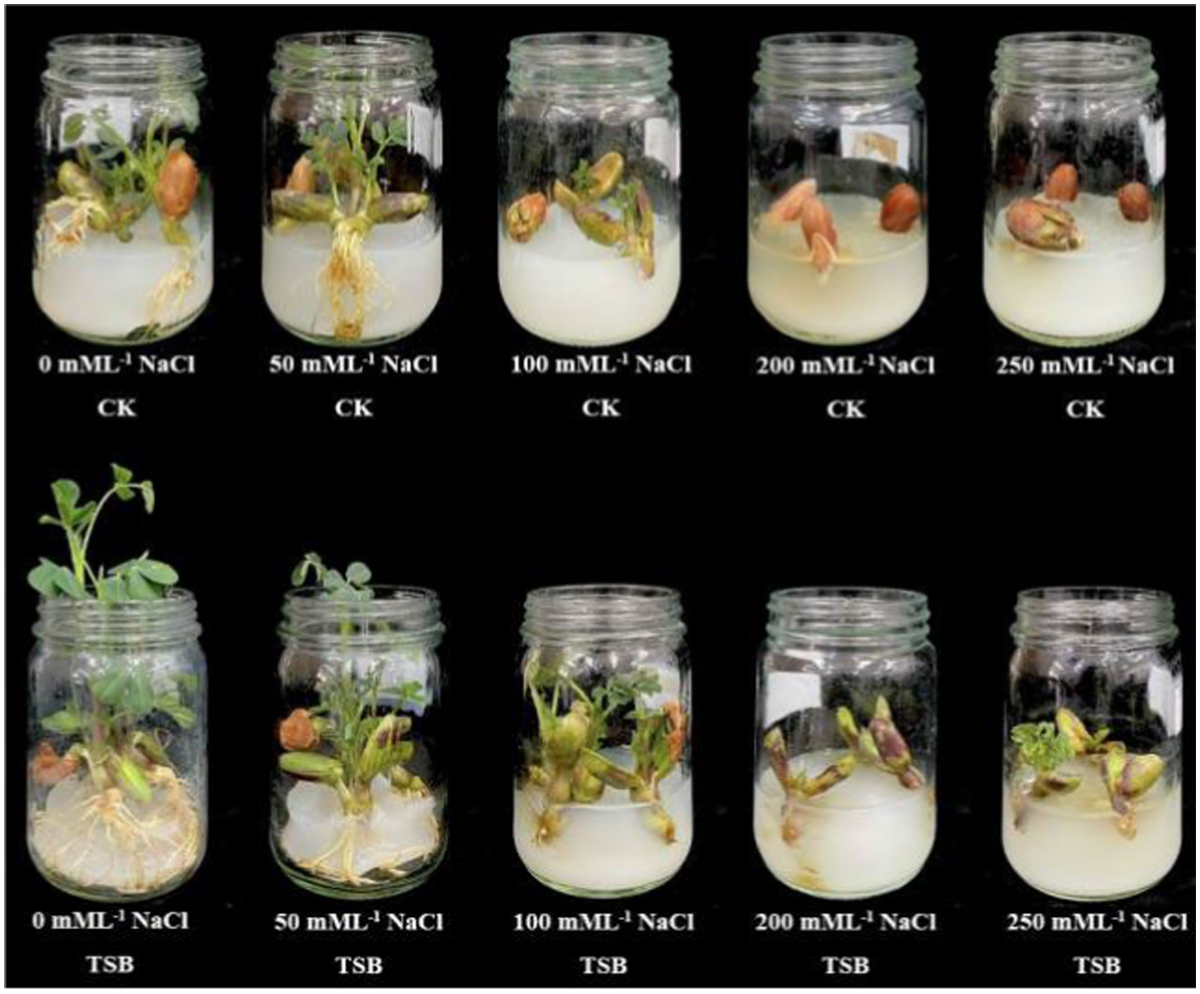
Peanut seed germination under different levels of salt (0 mM L^−1^, 50 mM L^−1^, 100 mM L^−1^, 200 mM L^−1^ and 250 mM L^−1^) without TSB (CK) and with TSB.

Peanut seedlings were then planted in plastic pots (height 10.9 cm, upper caliber 12.0 cm, bottom diameter 8.5 cm), each filled with 500.0 g of field soil. The experiment comprised four treatments, each performed in nine sessions: (1) CK (using the control with LB liquid medium only); (2) pots with TSB; (3) pots with only NaCl; and (4) pots with NaCl (200 mM L^−1^) and TSB. The bacterial culture was applied as a solution containing NDJ1, NDJ40, and NDY2 at a concentration of 1 × 10^9^ cfu mL^−1^. We selection of a 200 mM L^−1^ of NaCl concentration was based on optimal seedling growth in the growth medium (Figure 1). Under natural light conditions, the temperature was 28 ± 5°C (day/night, 14/10 h) and humidity was maintained at approximately 80 ± 5%. After 7 days, seedlings with uniform growth were selected and transferred to the pots, and salt stress was administered to the seedlings at 5-day intervals, following the protocols outlined for the four different treatment groups.

Plant samples for various analyses were collected after 30 days of treatment. Plant parameters, including root length, stem length, leaf area, DW and FW of peanuts, were measured as relevant indicators. Before sampling, chlorophyll levels in the leaves were analyzed using the KONICA MINOLTA Soil and Plant Analyzer Development (SPAD)-502 PLUS. All measurements were taken on the fourth lower leaf of each seedling.

The plant material was cut and weighed using an analytical balance to obtain the FW. Subsequently, the plant material was placed in an oven at 100°C–105°C for 10 min to remove any green color, followed by reducing the oven temperature to approximately 70°C–80°C until a constant weight was achieved. Finally, the analytical balance was employed to measure the DW. After sampling peanuts at the seedling stage, the samples were promptly washed, dried, and ground to extract the supernatant as an enzyme source. Following the manufacturer’s protocol, enzymes such as SOD, POD, CAT, PPO, and MDA were extracted using kits purchased from Novogene Co. Ltd. The enzyme activity was calculated by measuring the luminosity value with an enzyme marker according to the provided instructions.

POD activity was assessed spectrophotometrically using 190.0 μL of 4.0 mM guaiacol solution and 10.0 μL of the initial supernatant (enzyme extract), with a reaction time of 1 min. The estimation of POD activity relied on the production of guaiacol, the reaction product. POD (ΔA_470_/g/min) can be calculated using the formula: POD = (ΔA_470_ × *Vt*)/(*W* × *Vs* × *t*), where ΔA_470_ represents the change in absorbance during the reaction time, *t* denotes the duration of the reaction (min), *W* signifies the fresh weight of leaves (g), *Vt* stands for the total volume of the extracted enzyme solution (mL), and *Vs* indicates the volume of enzyme solution utilized for the determination (mL). MDA content was determined via the thiobarbituric acid (TBA) method. The supernatant was reacted with a 0.5% TBA solution and 20% aqueous trichloroacetic acid. This reaction mixture was incubated in a water bath for 30 min, and the optical density of the solution was measured at 532 nm. A molar extinction coefficient of 1.55 × 10^−3^ (L M^−1^ cm^−1^) was utilized to calculate the MDA content (nM g^−1^). The MDA content (nM/g) is determined using the formula: MDA = 32.3 × ΔA ÷ *W*, where ΔA represents the change in absorbance over the reaction time, calculated as the difference between A_532_ and A_600_. Here, *W* signifies the fresh weight of leaves (g). SOD activity was evaluated using the nitrogen blue tetrazolium (NBT) reduction method. This involved adding 70.0 μL of 0.1 M methionine, 80.0 μL of 2.0 mM NBT, and 10.0 μL of 0.012 mM riboflavin to 20.0 μL of supernatant. After allowing the solution to stand for 30 min away from light, the absorbance was measured at 450 nm. One SOD unit was defined as the amount of enzyme necessary to inhibit 50% of the nitroblue tetrazolium photoreaction compared to test tubes lacking plant extracts. SOD (U/g) is calculated as (Ack - AE) × *Vt*/Ack/0.5/*W*/*Vs*, with Ack as the absorbance of illuminated control tubes with buffer instead of enzyme solution, and AE as the absorbance of sample tubes. *W* represents the fresh weight of leaves (g), *Vt* is the total volume of the extracted enzyme solution (mL), and *Vs* indicates the volume of enzyme solution used for determination (mL). The determination of PPO activity was carried out using the catechol method as described in the reference of Xiao et al. (2024). PPO (ΔA_420_/min/g) is calculated using the formula: PPO = 400 × ΔA_420_ ÷ *W*, where ΔA_420_ represents the change in absorbance over the reaction time, and *W* denotes the fresh weight of leaves (g).

CAT activity was determined by UV spectrophotometry. A sample supernatant of 10.0 μL was mixed immediately with 190.0 μL of the working solution, and the initial absorbance value (A1) at 510 nm was recorded, along with the absorbance value after 5 min (A2). Calculation ΔA = A1 – A2. CAT activity was determined by measuring the remaining amount of H_2_O_2_ (Du et al., 2024). Specifically, 10.0 μL of CAT was added to a 0.033 M H_2_O_2_ solution and allowed to react at 25°C for 5 min. The reaction was halted by adding 290.0 μL of 30% H_2_SO_4_, and the absorbance of the solution was recorded at 510 nm. A unit (U) of CAT activity represented the amount of CAT required to catalyze 1.0 μM of H_2_O_2_ in 1 min under specific reaction conditions. CAT (μM/g/min) is calculated using the formula: CAT = 141.6 × (ΔA_510_ + 0.0137) ÷ *W*, where ΔA_510_ is the absorbance change during the reaction time, and *W* is the fresh weight of leaves (g).

### Transcriptome analysis

Fresh peanut roots were collected, and washed with distilled water, and the surface tissue was thoroughly dried. Each sample, weighing approximately 500.0 mg, was then carefully placed into a 50.0 mL Eppendorf Tube, with three tubes of samples utilized per treatment. Subsequently, all samples underwent rapid freezing in liquid nitrogen, followed by storage in a −80°C deep freezer refrigerator. For transportation, the samples were packed with dry ice and sent to the testing facility. The library preparation and RNA-sequencing procedures were conducted at Chengqi Medical (Shenzhen) Technology Co. Ltd. Detailed information regarding the RNA-seq data can be found in the reference genome and gene model annotation files of *A. hypogaea* which were directly downloaded from the NCBI genome website. An index of the reference genome was established using hisat2-2.0.4. Differentially expressed genes (DEGs) were identified based on Hochberg-adjusted *p*-values (FDR) < 0.05, with a | log2 (Fold change) | >1 threshold. The GO term pathways were determined with FDR < 0.05 and were deemed significantly altered, obtaining GO annotations based on biological processes. *A. hypogaea* was designated as the internal reference genome for this analysis.

### Statistical analysis

Tukey’s test was used for mean comparisons in instances of significant differences. Data derived from the experiment were analyzed at *p* < 0.05 using SPSS 26.0 software and presented as mean ± standard error (SE). A significant variations in peanut traits among different treatments were assessed through a two-way analysis of variance (ANOVA). The Fisher’s Least Significant Difference (LSD) test was applied to ascertain the statistical significance of differences between means. GraphPad Prism 9.0, Excel 2016, and Image-Pro Plus 6.0 were utilized for data processing, plotting, and statistical analysis. Adobe Photoshop 2020 and Adobe Illustrator 2021 were employed for image manipulation.

## Results

### Identification of strains

**Figure 2:**
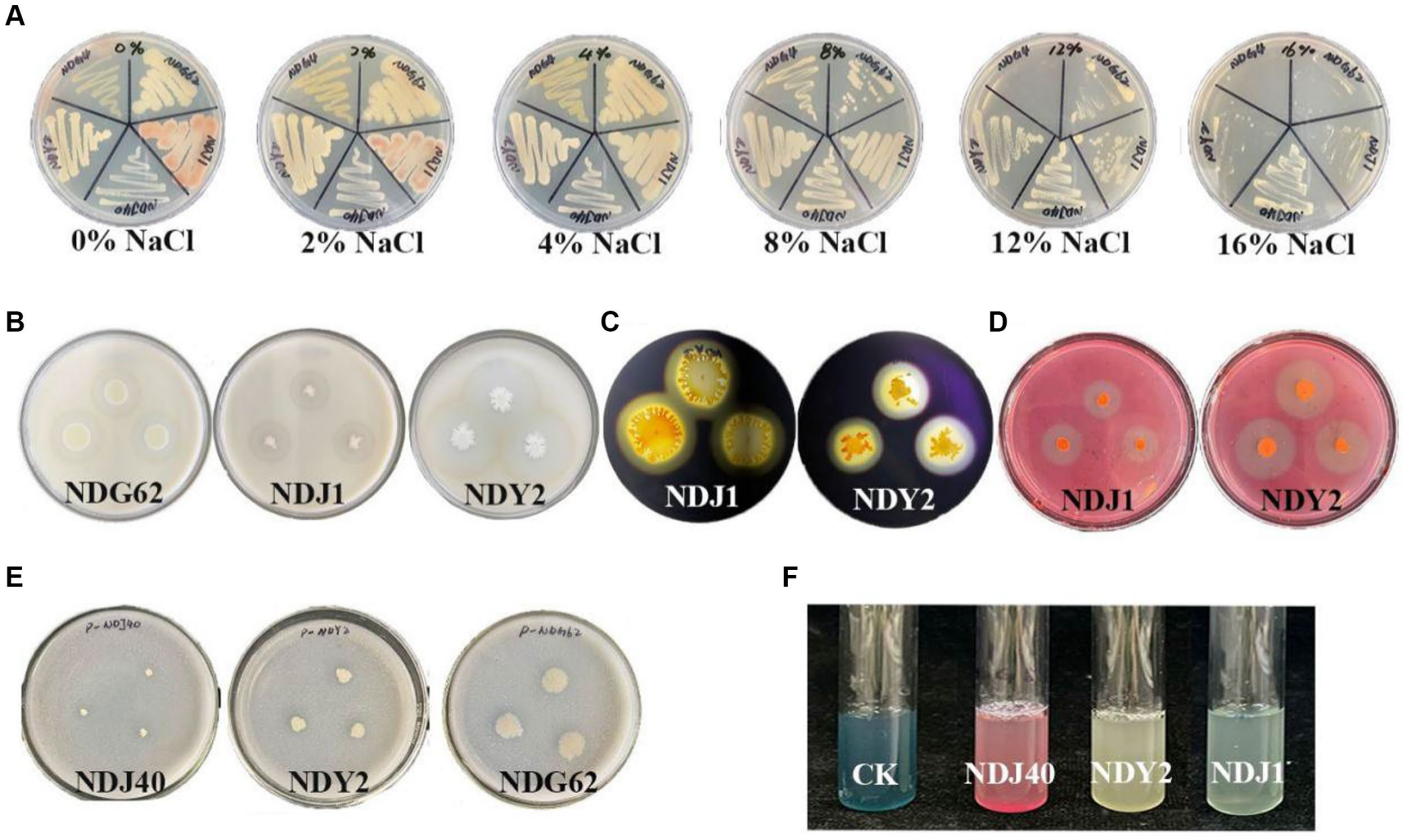
Salt tolerance and qualitative tests of different bacterial enzymes. **(A)** Salt stress. **(B)** Bacterial protease production. **(C)** Amylase producing. **(D)** Cellulase production. **(E)** Bacterial phosphate utilization. **(F)** Production of iron carriers.

A total of 22 endophytic bacteria were isolated and identified based on morphological features from *M. lutariariparius* (Supplementary Table S1). Out of these, 12 strains exhibited optimal growth at 4%, while five thrived at 8% (NaCl w/v) (Supplementary Table S2). Notably, only four strains NDJ1, NDJ40, NDY2, and NDG62 demonstrated tolerance to a 16% salt concentration. The colony color of NDJ1 at 4% NaCl concentration transitioned from pink to yellowish (Figure 2A).

The results of the physicochemical experiments conducted on the bacteria are presented in Supplementary Table S3. Protease activity was positively observed in NDG62, NDJ1, and NDY2, leading to the formation of clear hydrolytic zones (Figure 2B). Moreover, NDJ1 and NDY2 exhibited the ability to hydrolyze cellulose into glucose (Figure 2D) and demonstrated amylase production (Figure 2C). Notably, when NDG62, NDJ1, and NDJ40 strains were inoculated into the Phosphate Solubilization medium, a transparent circle formed around the colony, indicating phosphate solubilization activity (Figure 2E). Additionally, NDJ1, NDJ40, and NDY2 exhibited enhanced affinity of iron carriers for Fe (III), as detected by a colorimetric reaction due to chelation, as compared to controls (Figure 2F). The comprehensive results encompassing protease, cellulase, and amylase activities, as well as phosphorus solubilization and iron-carrier interactions, are detailed in Supplementary Table S3.

In addition, the 16S *rRNA* gene sequences of NDG62, NDJ1, NDJ40, and NDY2 exhibited similarities of 99.59, 99.90, 99.80, and 99.89%, respectively, to known bacterial strains: *Priestia aryabhattai* B8W22, *B. tequilensis* KCTC 13622, *S. epidermidis* NCTC 11047, *B. siamensis* KCTC 13613.

### Seed germination

As the salt concentration increased, the physiological indices of each treatment group exhibited a decreasing trend, suggesting that elevated salt stress poses a threat to peanut growth. Notably, among the four tested bacterial combinations, the “J1 + J40 + Y2” combination performed exceptionally well in promoting seedling growth at 250 mM L^−1^ (Table 1; Supplementary Table S4). In the TSB-only treatment, plant length and root length increased by 81.81 and 14.07%, respectively, compared to the CK without salt stress (Table 1). In contrast to CK treatments, NaCl_TSB treatments (100 mM L^−1^ NaCl) significantly (*p* < 0.01) increased stem and root lengths by more than 2.5-fold. At a 250 mM L^−1^ NaCl concentration, TSB facilitated plant growth even without roots, while in the control (CK), neither roots nor stems developed. Our findings strongly suggest that TSB possesses the ability to promote plant growth even under high salt conditions.

**Table 1:** Two-way ANOVA for the influence of TSB and CK on plant length, root length and biomass of peanut under different NaCl levels.

Treatment	Plant length (cm)		Root length (cm)		Plant biomass (g FW)		Plant biomass (g DW)	
	CK	TSB	CK	TSB	CK	TSB	CK	TSB
0 mM L^−1^ NaCl	9.79 ± 1.20	17.80 ± 1.50	4.12 ± 0.34	4.70 ± 0.59	3.93 ± 0.03	4.25 ± 0.33	1.03 ± 0.57	0.79 ± 0.11
50 mM L^−1^ NaCl	8.77 ± 2.02	11.42 ± 2.91	3.51 ± 1.14	4.08 ± 1.46	3.63 ± 0.49	3.66 ± 0.48	0.86 ± 0.17	0.85 ± 0.23
100 mM L^−1^ NaCl	2.30 ± 2.00	6.88 ± 0.66	0.64 ± 0.35	1.91 ± 0.41	1.93 ± 1.67	3.24 ± 0.25	0.56 ± 0.48	0.96 ± 0.31
200 mM L^−1^ NaCl	–	3.78 ± 0.31	1.23 ± 1.07	2.36 ± 0.45	–	2.23 ± 0.17	–	0.79 ± 0.09
250 mM L^−1^ NaCl	–	3.74 ± 0.56	–	–	–	2.16 ± 0.35	–	0.72 ± 0.07
ANOVA	*F*	*p*	*F*	*p*	*F*	*p*	*F*	*p*
TSB	79.63	***	35.68	***	32.21	***	11.55	***
Salt	73.43	***	6.77	**	30.35	***	23.93	***
TSB × salt	2.98	**	0.69	ns	4.29	**	8.78	***

Difference in various growth parameters and comparative analysis. Average ± standard error from three separate replicates (*n* = 3). Different letters mean significantly different based on *p* < 0.05. “*,” “**,” and “***” indicate significant differences at *p* < 0.1, *p* < 0.05, and *p* < 0.01, respectively, and “ns” represents no significant difference.

### Pot experiment

**Figure 3:**
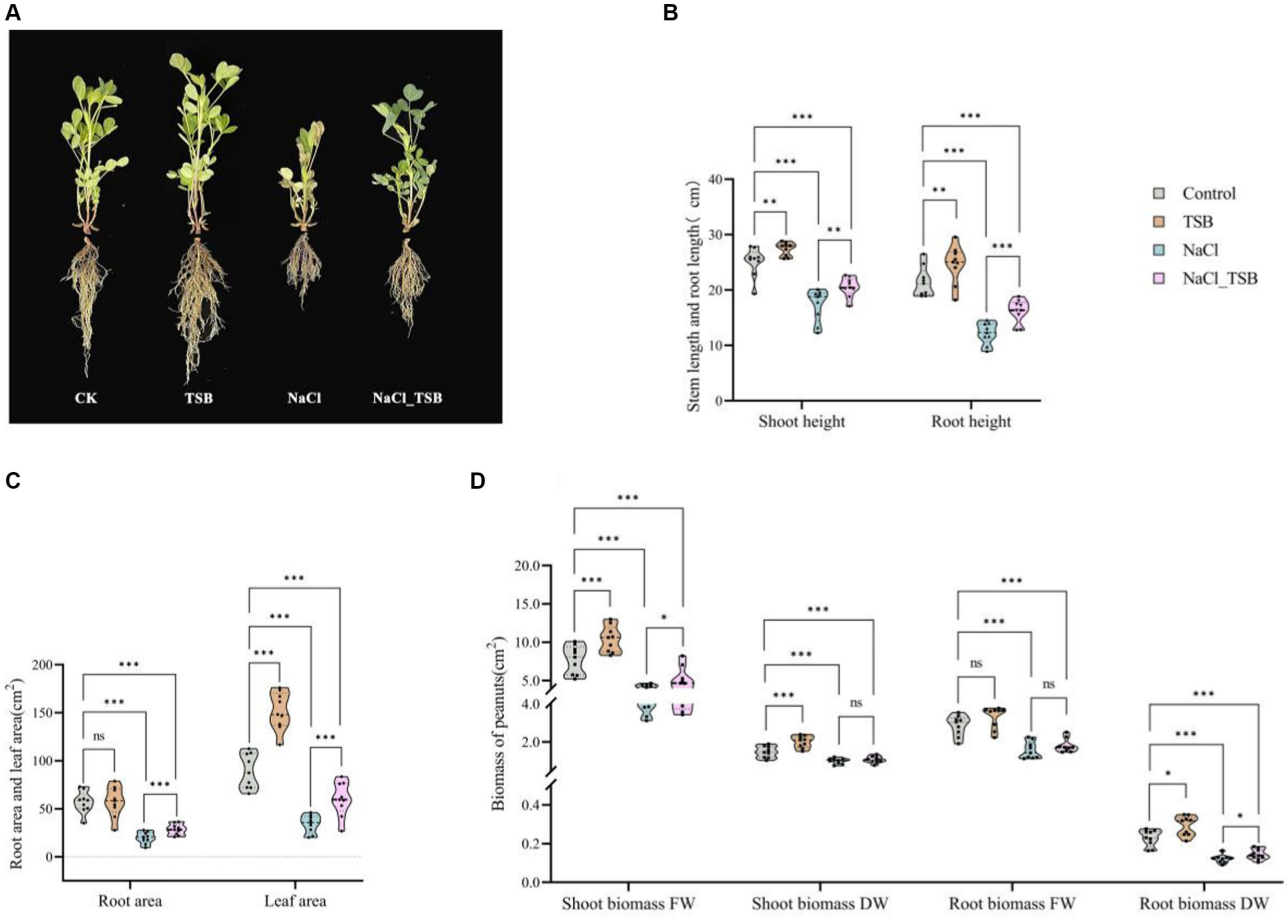
Plants growth in pots experiment under all treatments. **(A)** Pot test plot. **(B)** Stem and root height, **(C)** root area and Leaf area, **(D)** Shoot and root biomass of peanuts. Note that “*”, “**”, and “***” indicate significant differences at *p* < 0.1, *p* < 0.05, and *p* < 0.01, respectively, and “ns” represents no significant difference.

In the pot experiment, the exclusive application of TSB demonstrated a significant increase in all plant growth parameters compared to the CK, NaCl, and NaCl_TSB treatments (Figure 3A). Under normal conditions, peanuts treated with TSB exhibited greater height and stronger roots than those in the CK group, indicating that TSB plays a role in promoting peanut growth. Interestingly, a similar promotion of root growth by TSB was observed in peanuts subjected to salt stress conditions.

TSB treatment resulted in a significant increase in stem length, root length, and leaf area by 8.99, 14.55, and 70.12% (*p* < 0.05), respectively, compared to the control. Conversely, SFW, SDW, and RDW exhibited substantial increments of 34.93% (*p* < 0.01), 36.73% (*p* < 0.01), and 58.33% (*p* < 0.1), respectively (Figures 3B–D, Supplementary Table S5). Importantly, all physiological indices root length, stem length, root area, leaf area, and biomass were significantly lower in the NaCl treatment compared to CK (*p* < 0.01), indicating severe inhibition of peanut growth under 200 mM L^−1^ salt stress. NaCl_TSB treatment notably increased peanut stem length by 18.22%, root length by 33.58%, root area by 43.25%, and leaf area by 75.56%, compared to NaCl alone. Furthermore, both NaCl and TSB exhibited significant effects on various plant parameters (Supplementary Table S5). While the biomass of peanuts showed higher values for SDW and RFW, there was no significant difference in NaCl_TSB compared to the NaCl treatment. However, SFW and RDW saw significant increases of 27.06 and 16.66% (*p* < 0.1), respectively.

### The activity of POD, SOD, MDA, CAT, and PPO

**Figure 4:**
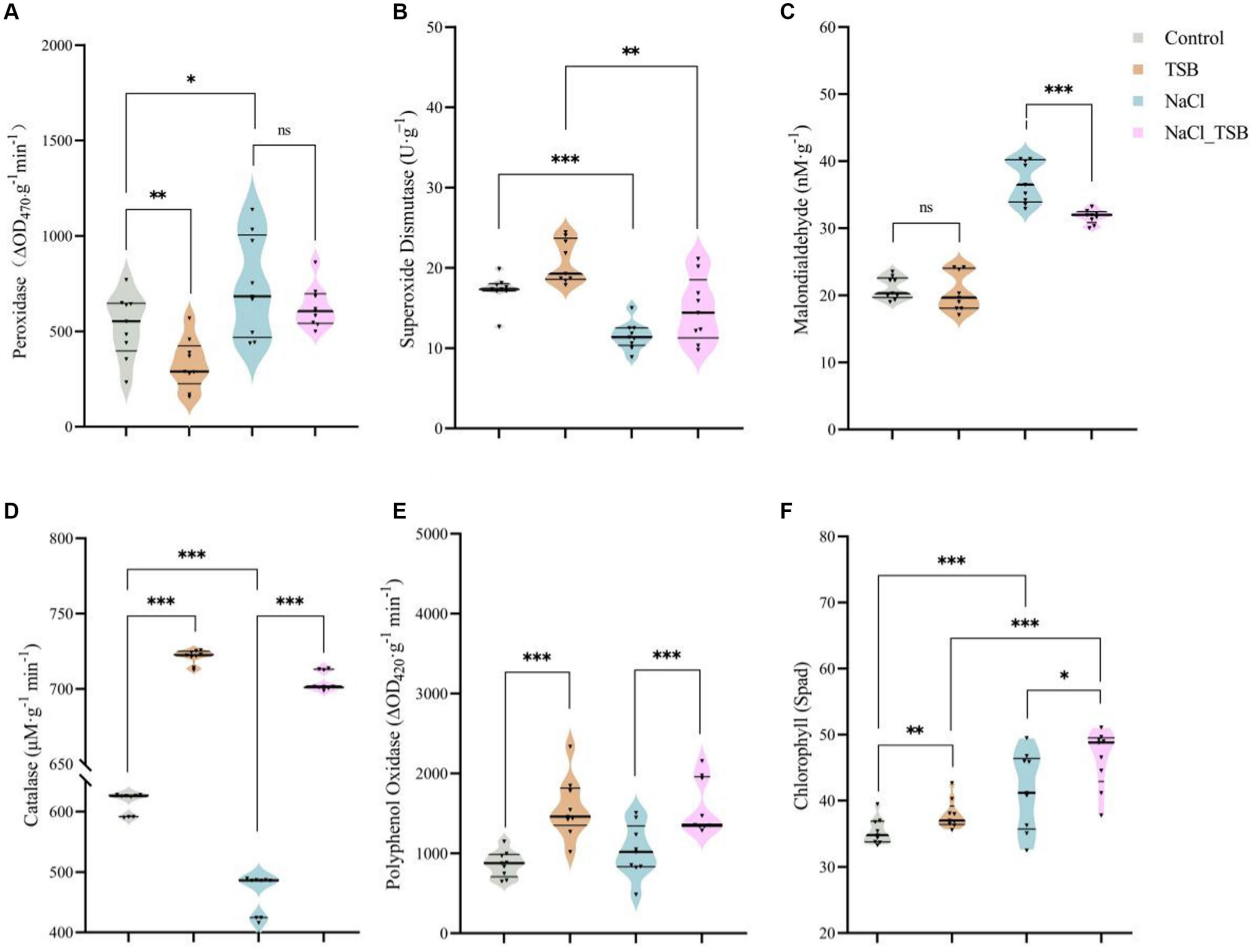
Plant enzymes under different treatments. The activity of **(A)** peroxidase, **(B)** superoxide dismutase, **(C)** malonic dialdehyde, **(D)** catalase, and **(E)** polyphenol oxidase. **(F)** SPAD values in peanuts. Note that “*,” “**,” and “***” indicate significant differences at *p* < 0.1, *p* < 0.05, and *p* < 0.01, respectively, and “ns” represents no significant difference.

Under salt stress, the enzyme activity of POD and MDA in peanuts significantly increased by 38.86 and 75.16%, respectively, compared to the control (CK) (Figures 4A,C). However, the addition of TSB treatment only marginally reduced the level of POD without reaching statistical significance. Importantly, the incorporation of TSB under salt stress led to a notable 16.51% decrease in MDA levels, mirroring the effect observed for POD. Interestingly, there was no significant difference in MDA levels under normal conditions with or without TSB addition (Figure 4C). These findings suggest that the addition of TSB mitigates salt stress by reducing the enzyme activities of both POD and MDA.

SOD and CAT exhibited significant decreases of 75.16 and 32.13%, respectively, in peanuts under salt stress compared to the CK. However, TSB demonstrated a notable alleviation of salt stress by enhancing the enzyme activities of SOD and CAT by 28.00 and 51.48%, respectively (Figures 4B,D). The activity of the PPO enzyme decreased under salt stress, whereas TSB significantly increased it (Figure 4E), suggesting that TSB aids peanuts in mitigating stress by boosting PPO activity. Two-way ANOVA results indicated that TSB treatment significantly affected the activities of five enzymes in peanuts, including SOD, POD, CAT, PPO, and MDA. In addition to the PPO enzyme, the activities of the other enzymes were also found to be associated with salinity stress (Supplementary Table S6).

### Chlorophyll-related SPAD values of peanut

Salt stress altered the color of peanut leaves, turning them from green to a darker shade compared to the control (CK), leading to corresponding changes in peanut SPAD values (Figure 3A). Moreover, a two-way ANOVA analysis identified salt pressure and TSB treatments as crucial factors influencing peanut SPAD values (Supplementary Table S6). The SPAD meter-measured chlorophyll content revealed a significant 17.36% increase (*p* < 0.01) in the chlorophyll SPAD index in NaCl-treated plants compared to the control. Additionally, TSB treatment resulted in a notable 7.11% increase (*p* < 0.05) in the chlorophyll SPAD index compared to the control, while the NaCl_TSB combination showed an 11.76% increase (*p* < 0.1) compared to NaCl alone. This suggests that the application of TSB to peanuts, whether under salt stress conditions or not, led to an elevation in the chlorophyll SPAD index.

### Transcription and expression analysis in peanut roots

In this experiment, a total of 12 cDNA libraries were prepared to analyze the alterations in peanut seedlings following TSB treatment under both salt stress and non-stress conditions. During this phase, clean reads were acquired by removing raw reads containing adapters, poly-N sequences, or exhibiting low quality. Overall, 627,997,748 raw reads were generated from 12 cDNA libraries, and after the cleaning process, 614,906,794 (97.91%) clean reads were obtained. Additionally, the average Q20, Q30, and GC content of the clean data were calculated at 97.92, 94.02, and 44.13%, respectively (Supplementary Table S7). Comparison of the clean reads from each sample with the assigned reference genome revealed matching efficiencies ranging from 92.71 to 97.05%. Furthermore, it yielded 89.71 to 94.85% uniquely mapped reads to the reference genome (Supplementary Table S8).

**Figure 5:**
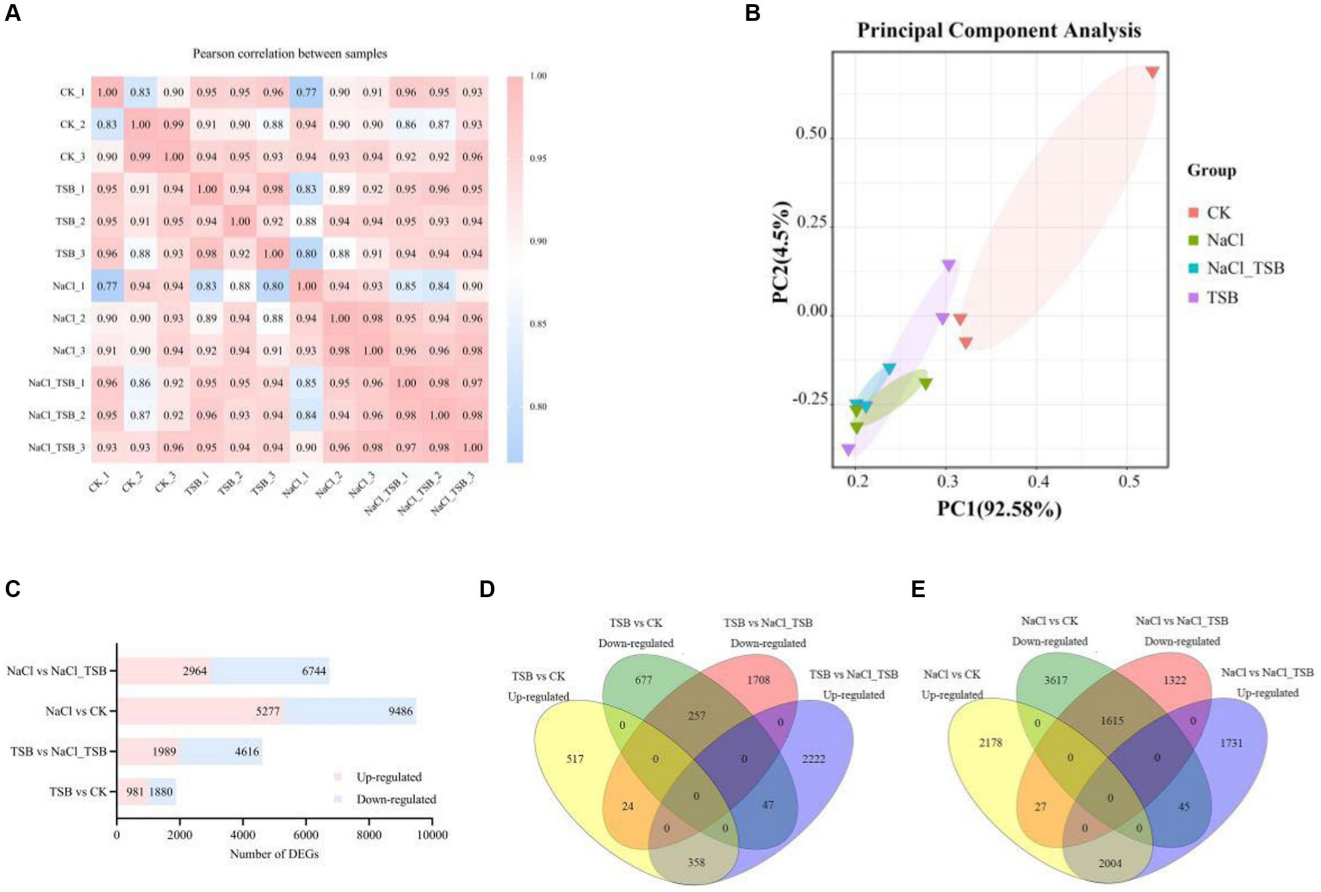
Heatmaps and Venn diagrams of differentially expressed genes of TSB in peanuts under normal and salt stress conditions. **(A)** Sample-sample correlation heatmap of 12 samples treated with 0 and 200 mM L^−1^ NaCl. **(B)** PCA plot. Different ellipses show 95% confidence intervals for samples from four treatments. **(C)** Gene up-and down-regulation between groups. **(D)** Venn diagram of up-regulated and down-regulated genes between groups.

To understand the difference of transcriptome between different treatments, the similarity coefficient between the four treated samples is very high as shown in Figure 5A. Based on the distribution of the total sample (PCA analysis) can visualize the characteristics of each sample and understand the correlation and differences between them. As shown in Figure 5B, the CK group was more discrete in distribution relative to the other groups, while the TSB treatment was a little more clustered relative to CK. It indicates that the application of TSB under no salt stress caused changes in the structure of peanut principal components. Analyzing the treatment groups for salt stress revealed that the principal components of peanuts were closer together, probably because salt treatment made the differential gene expression in peanuts more similar.

### Differential gene expression after saline treatment

Differential expression analysis of two conditions was performed using the edgeR package (3.18.1). DEGs were determined by the standard of log2 Fold Change ≥ 1, and FDR ≤ 0.05. Based on the RNA-Seq data, we identified 2,861, 6,605, 14,763, and 9,708 genes in the comparative analyses of TSB vs. CK, TSB vs. NaCl_TSB, NaCl vs. CK, and NaCl vs. NaCl_TSB, respectively (Figure 5C). Noteworthy is the observation that in comparison to the 2,861 differential genes identified in TSB vs. CK, the count of differential genes in NaCl vs. CK was significantly augmented, reaching a total of 14,763 DGEs. This disparity suggests a substantial alteration in the peanut root transcriptome, indicative of a robust regulatory response to environmental stressors, thereby signifying the capacity of peanut roots to withstand the adverse impacts of the adapted environment.

Furthermore, the total number of up-and down-regulated genes in NaCl vs. NaCl _TSB was reduced by 78.03 and 40.65%, respectively (Figure 5C), compared with NaCl vs. CK. This reduction underscores the discernible impact of applying TSB to peanut plants under salt stress, implying a modulatory effect on the transcriptional expression of genes within the peanut inter-root environment.

To elucidate the impact of TSB on the inter-root transcriptome of peanut plants subjected to salt stress, we conducted a comprehensive analysis of the TSB vs. CK and TSB vs. NaCl _TSB groups employing Venn software. Notably, 615 DEGs were identified as co-expressed in both groups (Figure 5D), while 1,194 and 3,930 DEGs exhibited unique expression patterns in the TSB vs. CK and TSB vs. NaCl _TSB groups, respectively. Within this cohort of DEGs, 358 genes demonstrated significant up-regulation, whereas 257 genes exhibited significant down-regulation in both the TSB vs. CK and TSB vs. NaCl _TSB groups. This indicates that TSB treatment made a total of 615 genes significantly change after TSB treatment (Figure 5E).

### Differential gene expression in peanut under salt stress

**Figure 6:**
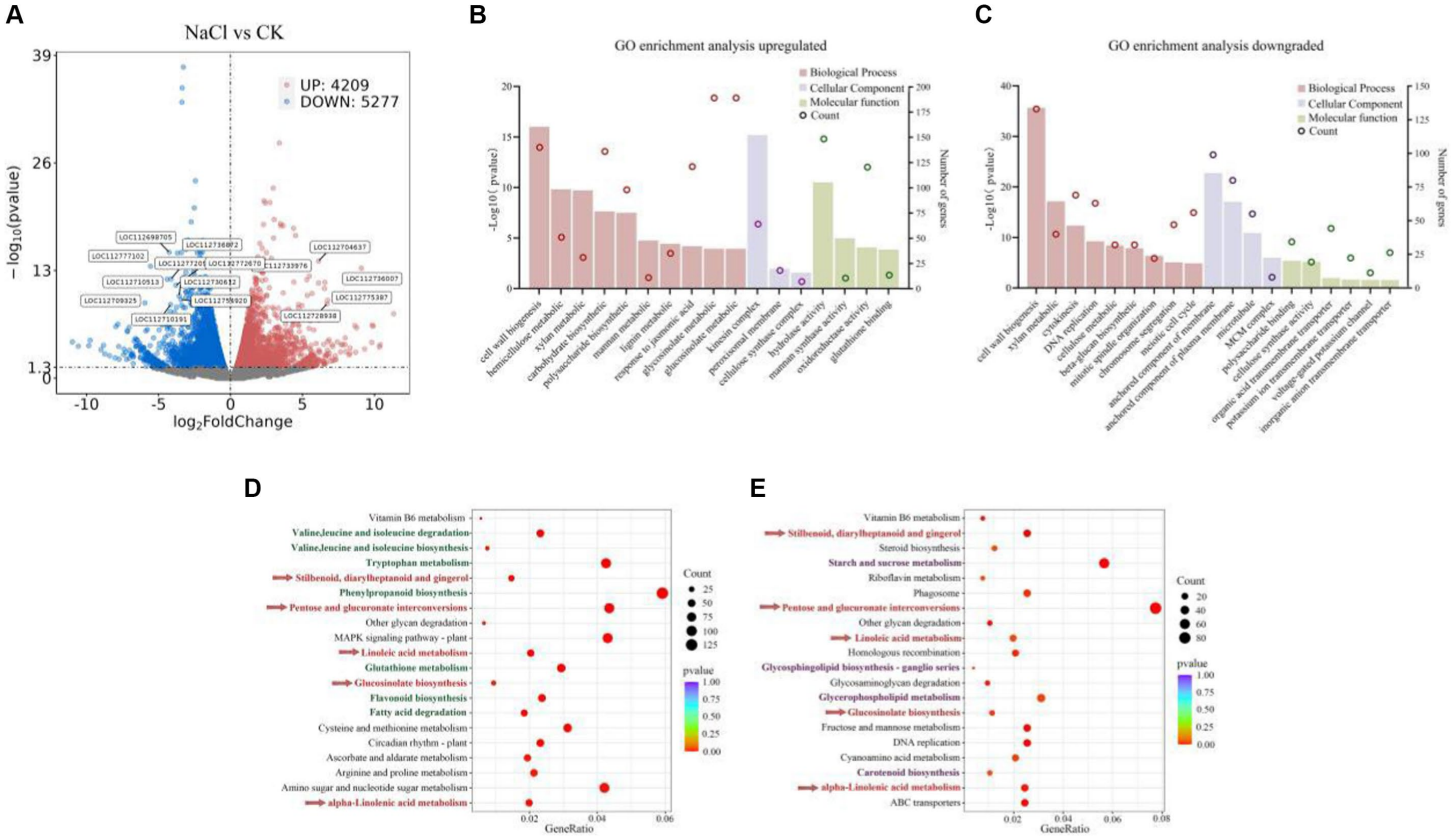
**(A)** Volcano plot of differentially expressed up and down regulated genes under salt stress. GO term enrichment of up-regulated **(B)** and down-regulated **(C)** genes of *A. hypogaea* under salt stress. Top 20 up **(D)** and down **(E)** enriched KEGG pathways of *A. hypogaea* (different colors indicate different profiles, corrected *p* value < 0.05).

To characterize the transcriptome of peanut roots under salt-stress, gene annotation and expression analysis detected 54,628 genes. These findings suggest that salt-tolerant peanut HY25 undergoes more activation at the transcriptional level when sensing salt stress signals, including 9,488 DEGs (| log2 fold-change | ≥ 1 and *q*-value ≤ 0.05) (4,209 up-regulated and 5,277 down-regulated) (Figure 6A).

As shown, peanut significantly down-regulated 1-deoxy-D-xylulose-5-phosphate synthase, chloroplastic (LOC112698705), a terpene synthase (LOC112772670) involved in the formation of plant terpenoids under salt stress. In addition, simultaneous significant down-regulation of ethylene-responsive transcription factor (LOC112710513), and lipoxygenase (LOC112710191), as they play an interacting role in plant physiological processes. Notably, salt stress significantly down-regulated the protein flowering locus (LOC112754920), which is associated with plant pro-flowering, as well as the disease resistance protein DSC1 (LOC112777102, LOC112709325). It suggests that the specific mechanism of action and the extent of interactions in peanut under abiotic stress may be influenced by many other factors, such as phytohormone enzyme activities, and down-regulation of transcription factors associated with physiological processes. In addition, salt stress in peanut upregulates signaling molecules regarding the enhancement of autoimmunity through autoregulation and protects cells from free radicals against environmental stress. Examples include the up-regulation of plant antimicrobial peptides (LOC112733976) to protect against pathogens, or the up-regulation of a series of monothiol glutathione (LOC112704637), specific mannose lectin (LOC112775387), which protects plant cells from free radical damage (Figure 6A).

GO enrichment analysis revealed that biological processes in peanut cells under salt stress were significantly enriched in genes related to cell wall responses, and in addition, lignin, a component of the cell wall, was also significantly enriched, suggesting that peanut maintains the composition and structure of the cell wall through the up-regulation of membrane plasmodesmata-related genes under salt stress, as the cell wall is the first barrier for plants to resist the adversity-stressed environment (Figure 6B). In addition, sugar metabolism genes were significantly up-regulated in peanut, including polysaccharides, xylans, mannans and glucosinolates as well as hemicellulose, and carbohydrate biosynthesis. Significant enrichment of hydrolytic enzymes acting on sugar groups was likewise found in molecular functions (Figure 6B). This may be due to the increased sugar metabolism in peanut during biological processes, which promotes hydrolases to catalyze hydrolysis reactions that break down large molecular compounds into small molecules to provide energy for the plant. We found that multiple biological processes related to the cell life cycle were significantly down-regulated and enriched in peanut in response to salt stress (Figure 6C), including cell division, DNA replication, mitosis and meiosis, and chromosome segregation. This suggests that the growth process of peanut cells under salt stress may be inhibited or slowed down, which in turn affects plant growth and development. Multiple ion channels and transporter proteins are significantly enriched in the molecular functions of peanut in response to salt stress processes, including organic acid transmembrane transporters, potassium ion transmembrane transporters and inorganic anion transmembrane transporters. This may affect plant water uptake and utilization, which in turn affects the plant’s immune response (Figure 6C).

KEGG enrichment analysis revealed that pathways linked to both up-and down-regulation, influenced by salt stress in peanuts, encompassed pentose and glucuronide interconversion, as well as glucosinolate biosynthesis (Figures 6D,E). These pathways collectively play a crucial role in regulating energy metabolism and substance synthesis in peanuts. Furthermore, linoleic acid metabolism exhibited significant enrichment through the modulation of pathways associated with plant defense, including the biosynthesis of stilbenes, diarylheptanes, and gingerols. This observation implies that peanut biosynthesis employs multiple enzyme-catalyzed reactions to mount a defense against external environmental threats through these intricate pathways (Figures 6D,E).

Illustrated in Figure 6D, a cascade of amino acid metabolic pathways, including tryptophan, valine, leucine, and isoleucine, alongside the pathways governing phenylpropanoid and flavonoid biosynthesis, represent specialized metabolites generated by plants during their normal growth to adapt to adverse environments. These metabolites play a pivotal role in promoting cell division and mitigating the physiological impacts of oxidative stress in plants. Glycerophospholipids and glycosphingolipids, crucial constituents of cell membranes, contribute to peanut’s resilience by compensating for phospholipid losses during adversity; their down-regulation, as illustrated in Figure 6E, results in biofilm instability in peanuts under salt stress. Moreover, the accumulation of reactive oxygen species in peanut cell membranes during stress makes unsaturated fatty acids on the glycerol backbone susceptible to attack. To prevent a rise in membrane permeability, cells significantly upregulate fatty acid metabolic pathways. Simultaneously, the upregulation of the glutathione metabolic pathway aids in scavenging reactive oxygen species, thereby mitigating cellular damage induced by membrane lipid peroxidation (Figure 6D).

The down-regulation of starch and sucrose metabolism appears to indicate a disruption in photosynthesis (Figure 6E). During photosynthesis, plants convert carbon dioxide and water into glucose, ultimately polymerizing it into starch as an energy store and a source of energy during periods without light. However, under salt stress, the starch and sucrose metabolic pathways were markedly down-regulated (Figure 6E), likely stemming from a decrease in the accumulation of substrates for photosynthesis and a reduction in the consumption of energetic substances produced by sucrose metabolism. Moreover, the concurrent down-regulation of carotenoid biosynthesis further substantiates the hindrance to photosynthesis.

### Evaluation of metabolic pathways potentially relevant to TSB promotion of salt tolerance in peanut

**Figure 7:**
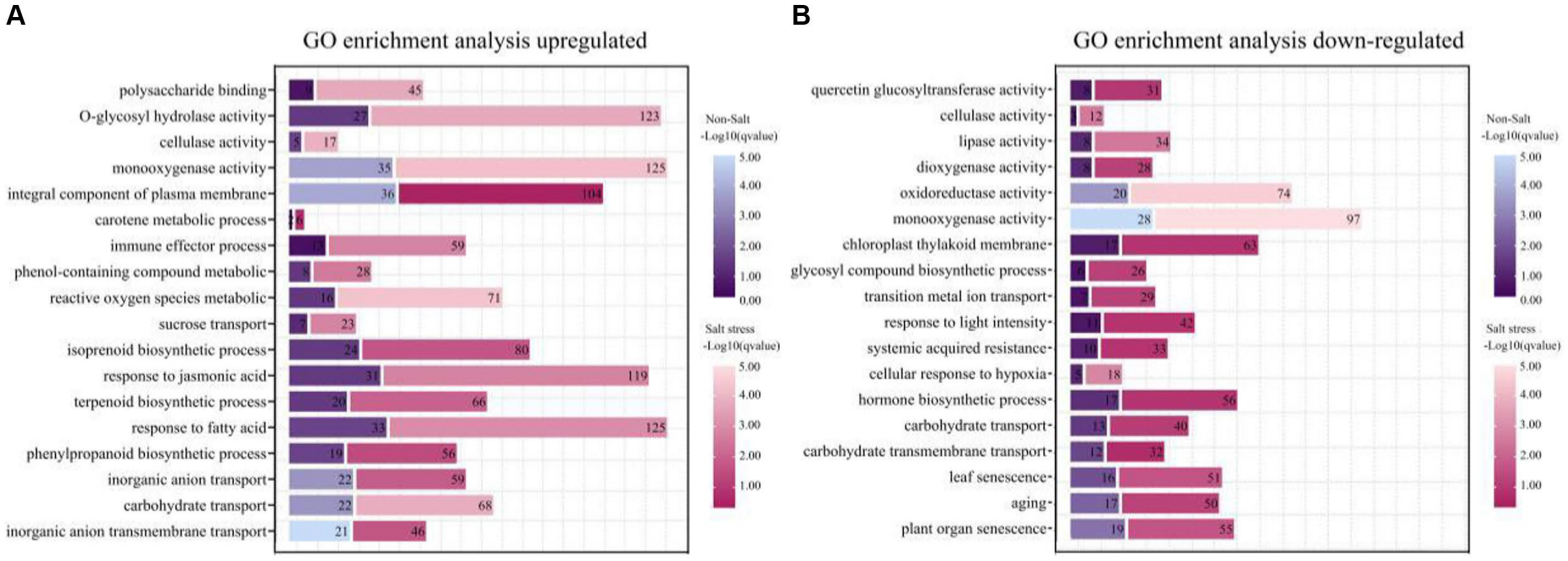
Functional enrichment of genes with different expression patterns in peanut in the presence or absence of bacterial solution and non-salt or salt stress (*p* < 0.05). GO functional classification analysis about genes of TSB vs. CK and NaCl_TSB vs. NaCl with different expression patterns. The top 18 entries were selected for statistical analysis. **(A)** up-regulation and **(B)** down-regulation.

Our scrutiny revealed that the application of saltwater induced a notable modulation in the expression of genes in peanuts, with an increased number of both up-and down-regulated genes compared to control conditions (Figures 7A,B). Particularly noteworthy was the 3.5-fold up-regulation of genes associated with polysaccharide binding and glycosyl compound hydrolase activities under salt stress, indicating that TSB application significantly influences the expression of genes related to sugar metabolism and enzyme activities (Figure 7A). Furthermore, our observations demonstrated that TSB treatment elevated the level of phenolic compound metabolism and catalyzed cellulase reactions, aligning with the experimental results elucidated earlier (Figures 2D, 4E). The up-regulation of carotenoid metabolic processes and isoprenoid biosynthesis, as depicted in Figure 7A, played a pivotal role in enhancing photosynthetic efficiency in peanut plants. However, it is crucial to note that the promotion of phenylacetone had an impact on isoprenoid biosynthesis within this process.

Under salt stress conditions, peanut cell membranes exhibit heightened sensitivity to oxidative reactions induced by reactive oxygen metabolism. In response to this stress, peanut bioprocesses undergo significant up-regulation of fatty acid metabolism, and the plasma membrane components play a crucial role in maintaining membrane stability. Notably, the expression of the jasmonic acid gene increased by 2.8-fold, providing clear evidence of the severe stress experienced by peanuts (Figure 7A). Conversely, salt stress led to a substantial down-regulation of genes associated with senescence, surpassing normal levels by more than 1.5 times when compared to the application of TSB under normal conditions (Figure 7B).

Furthermore, there was an observed modulation in carbohydrate transport, monooxygenase activity, and cellulase activity, with both up-regulated and down-regulated enrichments (Figures 7A,B). Additionally, key enzymes critical for plant growth, development, and response to environmental stresses, such as lipase, dioxygenase, and oxidoreductase, were down-regulated (Figure 7B). This down-regulation extended to genes involved in hormone biosynthesis, cellular response to hypoxia, and light intensity, rendering the plants more susceptible to environmental stresses and compromising their defensive mechanisms. The inhibition of photosynthesis further impacted plant growth and development.

### Identification of pathways for salt stress alleviation by TSB

**Figure 8:**
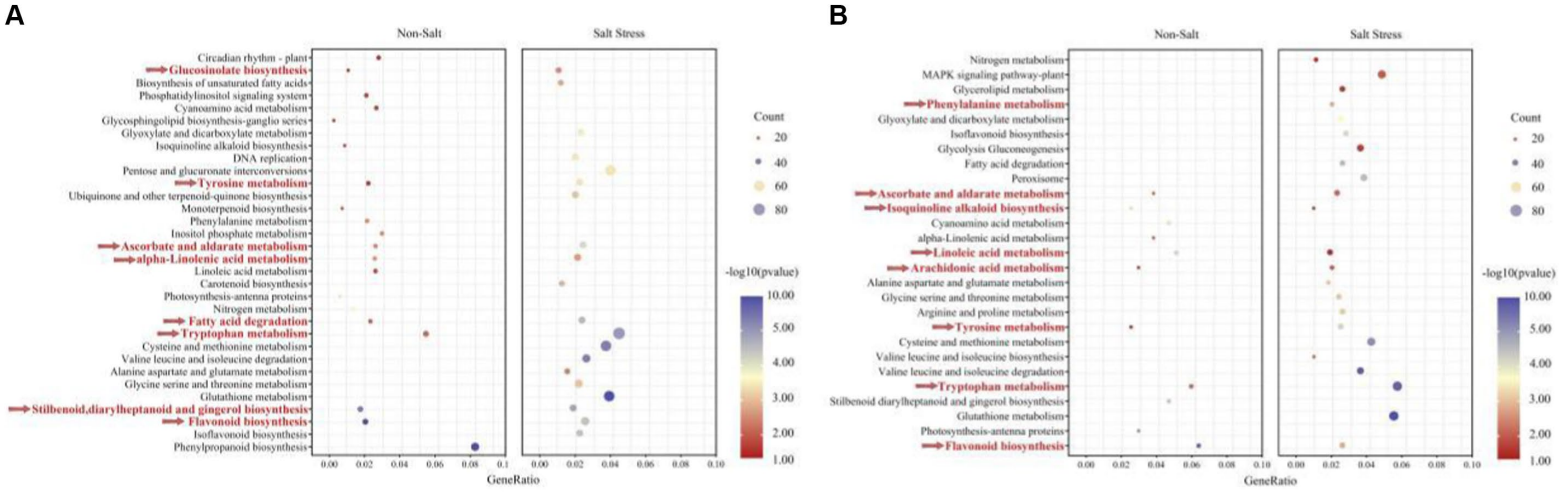
The KEGG enriched pathways of *A. hypogaea* **(A)** up-regulation; **(B)** down-regulation. Along with TSB contained the following two treatments: Non-stress; Salt stress. KEGG pathways enriched in temporal expression pattern profiles under salt-tolerate (different colors indicate different profiles, corrected *p* value < 0.05).

Our findings demonstrate that peanuts respond to TSB treatment by significantly up-regulating enriched pathways, specifically biosynthesis pathways associated with flavonoids, glucosinolates, astragalus, diarylheptanes, and gingerols. These pathways play a crucial role in promoting plant growth and alleviating stress. Furthermore, our analysis of pathways co-enriched in peanuts after TSB application, both in the presence and absence of salt stress, revealed a heightened significance and increased number of regulated genes under salt stress conditions (Figure 8A). Under normal conditions, TSB application triggered signaling in peanuts that positively up-regulated circadian rhythms, nitrogen metabolism, and photosynthetic pigment proteins, thereby orchestrating an environment conducive to plant growth and development (Figure 8A). Interestingly, biosynthesis of carotenoids in peanuts was inhibited under salt stress conditions (Figure 6E). However, TSB application under salt stress led to a significant up-regulation in the biosynthesis of carotenoids, a co-pigment crucial for photosynthesis (Figure 8A). This suggests that TSB effectively stimulates and enhances the photosynthetic efficiency of peanuts, consequently influencing various aspects of sugar metabolism and other physiological processes within the plant. Our study highlights the multifaceted effects of TSB on peanut metabolism, shedding light on its potential as a stress-alleviating and growth-promoting agent in agricultural practices.

As depicted in Figure 8A, the application of TSB under salt stress conditions resulted in distinctive metabolic changes compared to normal conditions. Specifically, the metabolic pathways of amino acids (tryptophan, glycine, serine, threonine, proline, aspartic acid, glutamic acid, cysteine, and methionine) exhibited a significant and enriched up-regulation. These amino acids not only play crucial roles in protein synthesis but also contribute to key amino acids involved in plant growth, development, and defense responses.

In our investigation, we observed a consistent down-regulation of the flavonoid biosynthesis pathway alongside the up-regulated enrichment pathway (Figures 8A,B). The number of genes up-regulated and enriched by TSB application was 73.77% higher than those down-regulated in the absence of stress. However, in the presence of salt stress, this percentage increased to 57.69%, with a higher significance in gene expression. Flavonoids, known for their efficiency in antioxidant and free radical scavenging, displayed increased expression under salt stress, indicating a robust resistance mechanism in peanuts. Remarkably, TSB application reduced the number of differentially up-regulated genes in the flavonoid biosynthesis pathway by 21.95% (Figures 6D, 8A), suggesting that TSB alleviates oxidative stress by modulating the synthesis of flavonoid compounds.

Contrary to the up-regulated pathways, arachidonic acid metabolism, along with ascorbic and aldolic acid, tyrosine, and tryptophan metabolism, showed significant down-regulation. Under salt stress, TSB stimulated peanuts to protect cells from oxidative damage during growth and development by regulating glutathione metabolism (Figure 8B). Additionally, amino acids directly associated with the glutathione metabolic pathways (glutamate, cysteine, and glycine), peroxisomes, and others displayed both up-and down-regulation, highlighting the intricate balance orchestrated by TSB in mitigating oxidative stress and fostering plant resilience. These findings provide valuable insights into the multifaceted regulatory role of TSB in modulating key metabolic pathways under stress conditions.

## Discussion

### TSB for efficient enhanced peanut development in saline soil

In this study, we have discerned that microorganisms play a pivotal role in mitigating the detrimental effects of salt stress by fostering plant development, enhancing plant resistance, and modulating phytohormone levels. Specifically, application of a mixture comprising three bacteria (*B. tequilensis*, *S. epidermidis* and *B. siamensis*) to peanuts has proven effective in alleviating salt stress. These findings align with prior research affirming the salt tolerance enhancement and growth-promoting properties of *B. tequilensis* and *B. siamensis* in abiotic stress conditions, ultimately leading to diminished crop losses (Shin et al., 2019; Haroon et al., 2022, 2023). In line with our anticipated outcomes, the screening of microorganisms influencing plant growth and phytoremediation, coupled with their application to peanuts in saline soils, holds promise for enhancing adaptability and boosting remediation efficiency.

### Substances assisting peanuts to withstand salinity

It is plausible that the extracellular secretions of microorganisms play a pivotal protective role. *S. epidermidis*, as reported by Chaudhry and Patil (2016), has been recognized for promoting plant germination and resistance through hydrolytic enzyme activity, iron carrier production, and volatile synthesis, thereby inhibiting phytopathogenic fungi. Importantly, soil salinization often leads to iron deficiency in various plant species, and the iron carriers secreted by microorganisms emerge as a valuable source for supplementing iron supply (Feng et al., 2023). Under biotic or abiotic stress conditions, H_2_O_2_ reacts with intracellular Fe (II) to generate hydroxyl radicals (−OH) (Paul et al., 2022), which, when entering the cytoplasm, can inflict damage on DNA structure. However, it has been reported that ferritin inhibits this reaction (Zhu et al., 2023), thus revealing a well-characterized mechanism for bacteria to produce iron carriers. In the endosphere, phosphorus-solubilizing bacteria *S. epidermidis* and *B. siamensis* immobilize available phosphorus from the soil into their cells (Bargaz et al., 2021), thereby enhancing the efficient utilization of nutritional resources by peanut roots under salt stress.

Proteases, by hydrolyzing H_2_O_2_, reduce cell death, forming bioactive peptides with antioxidant properties (Oh et al., 2019). Investigations have revealed that protein hydrolysis products from peas exhibit diverse free radical scavenging, linoleic acid peroxidation inhibition, and cholinesterase inhibiting activities (Asen and Aluko, 2022). Notably, linolenic acid is catalyzed by plant deoxygenase (LOX) to form hydrogen peroxide derivatives (e.g., aldehydes, ketones) and to derive jasmonic acid and other plant-damaging oxyphospholipids (Lung et al., 2022). Consequently, the use of protease-producing bacteria in saline soil could stimulate the jasmonate signaling pathway in peanuts, enabling plants to respond effectively to stress.

To date, research has conclusively shown that only microorganisms possess the ability to convert cellulose into sugars. In the presence of cellulase, cellulose breaks down into cellobiose, which further undergoes continuous breakdown into glucose, providing essential energy to the host (Li et al., 2023). Moreover, cellulose constitutes a crucial component of plant cell walls (Mahdi et al., 2021), and microorganisms can elevate the expression of cellulase.

### Effect of TSB on antioxidant enzyme activities in peanuts

The introduction of TSB in the presence of NaCl resulted in an augmentation of SOD and CAT activities, effectively scavenging superoxide and increasing H_2_O_2_ levels. However, the diminished levels of POD were insufficient to counteract the accumulated H_2_O_2_, ultimately disrupting the leaf’s photosynthetic system (Figure 4). As SOD activity rises, it facilitates the conversion of H_2_O_2_, with subsequent scavenging by CAT to convert it into water and oxygen. Nevertheless, under salt stress, there was a reduction in SOD and CAT activities in the leaf (Figure 4), consistent with findings that salt treatment significantly down-regulated the relative expression levels of antioxidant enzymes (SOD, POD, and CAT) in cotton leaves after 14 days of exposure to salt (Li et al., 2022). According to reports, phenolic compounds synthesized through the phenylpropanoid metabolic pathway, such as cresolase and catecholase, undergo oxidation to form quinones catalyzed by PPO. Subsequent non-enzymatic reactions result in the polymerization of these compounds into melanin as a key protective substance (Llorente et al., 2014; Tilley et al., 2023).

Interestingly, compounds such as OH^−^, H^−^_2_O_2_ and O_2_- in chloroplasts contribute to increased lipid peroxidation, with MDA serving as a marker for determination. MDA levels increased with escalating salt stress (Kutlu and Gulmezoglu, 2023). However, the MDA content in TSB-inoculated plants under saline conditions was lower (Figure 4C), suggesting a reduced accumulation of ROS and mitigated membrane damage. Our results are consistent with higher MDA enzyme activities observed after prior inoculation with *Glutamicibacter* sp. and *Pseudomonas* sp. compared to uninoculated salt-stressed *S. fruticosa* plants (Hidri et al., 2022).

### TSB improved photosynthetic pigments in peanuts under salt stress

Salt stress exerts inhibitory effects on the production of photosynthetic pigments, which are crucial for the vital processes of plants (Ullah et al., 2023). Interestingly, our results diverged from prior experiments by El-Maboud et al. (2023), it was demonstrated that in *J. rigidus*, exposure to 200 mM NaCl over 30-days period led to a notable increase of 1.5-fold in chlorophyll a, 1.58-fold in chlorophyll b, 1.52-fold in total chlorophyll, and 1.46-fold in carotenoids compared to the control. Similarly, Jiang et al. (2022) revealed that salt stress influences the content of additional pigments in peanuts, such as carotenoids and flavonoids, subsequently impacting leaf coloration. This supports the argument that salt stress might inhibit the activity of chlorophyllase in plants, leading to an elevation in leaf chlorophyll content (Li et al., 2015; Siddiqui et al., 2020).

### TSB enhanced plant oxidative and defense related pathways

In fact, diminished antioxidant enzyme activity may hinder the organism’s ability to effectively scavenge oxidation products, such as free radicals, leading to cellular damage and organ dysfunction (Angon et al., 2022). Intriguingly, our results underscore that TSB plays a pivotal role in facilitating the growth advantage of salt-tolerant peanuts in salt-stressed environments. Furthermore, we observed that genes related to amino acid metabolic pathways were significantly upregulated in peanuts treated with TSB under salt stress (Figure 8A), potentially aiding in preserving protein reserves in peanuts (Ahmed et al., 2022; Song et al., 2023). Similarly, pathways such as glutathione metabolism, ascorbate, and aldarate metabolism exhibited upregulation in response to TSB treatment (Figures 8A,B). Glutathione metabolism emerges as a pivotal pathway for plant survival, as glutathione plays a crucial role in the GSH-ascorbate redox system, effectively mitigating hydrogen peroxide toxicity (Banik and Bhattacharjee, 2020; Kopczewski et al., 2020). This highlights the potential of using high-salt-tolerant bacteria to activate plant defenses, enhance yields, and confer stress resistance.

**Figure 9:**
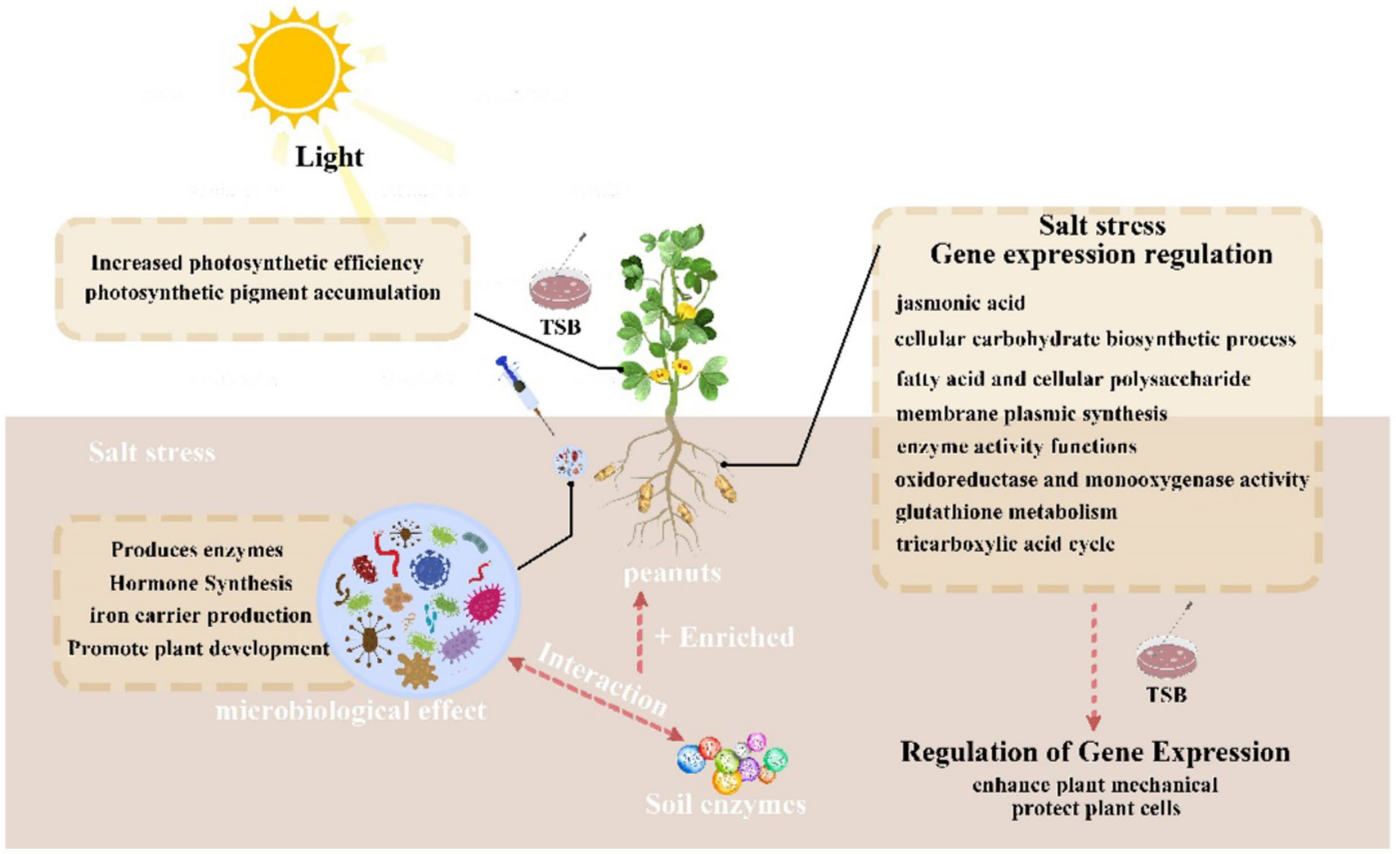
Summary graph.

Our study revealed that TSB-induced upregulation of fatty acid metabolism enables peanuts to maintain a normal intracellular environment under saline conditions, subsequently stimulating the tricarboxylic acid cycle (Figure 9). This cascade effect leads to increased synthesis and accumulation of various organic acids, contributing to the maintenance of healthy peanut tissues. Additionally, fatty acids, closely linked to the tricarboxylic acid cycle, play a pivotal role in energy storage and serve as biofilm components (Beebe et al., 2020; Choi et al., 2021; Kaestner et al., 2021). Interestingly, the jasmonate signaling pathway not only stimulates membrane composition but may also enhance root morphology, linoleic acid metabolism, and phytohormone signaling pathways. These results align with previous research demonstrating that wheat supplemented with exogenous jasmonic acid regulated the expression of many DEGs involved in phytohormone biosynthesis, including abscisic acid, jasmonic acid, and salicylic acid (Zhu et al., 2022). Additionally, jasmonic acid plays a crucial role in plant defense against herbivores and response to harsh environmental conditions and various abiotic and biotic challenges (Garcia-Abellan et al., 2017).

It is worth noting that most artificial microbial consortia encounter issues of instability and inefficiency due to growth competition and metabolite incompatibility. The applications discussed in this study have predominantly yielded positive results under controlled conditions. Therefore, we suggest that future research should expand to investigate bacterial stability in real-world production applications, especially in saline soils.

## Conclusion

Our findings offer valuable insights into understanding the salt stress tolerance mechanisms in peanuts facilitated by salt-tolerant bacterial combinations, specifically composed of *B. tequilensis*, *S. epidermidis*, and *B. siamensis.* This research lays a foundation for the comprehensive analysis of microbial genes and molecules involved in the development of salt stress resistance in plants. However, it is imperative to acknowledge that further studies are warranted to delve into the intricate mechanisms and genetic underpinnings of microbial-mediated mitigation of salt stress. Such endeavors are crucial for establishing a more robust and reliable foundation for practical applications in agriculture and environmental management.

## Data availability statement

The datasets presented in this study can be found in the online repository: https://www.ncbi.nlm.nih.gov/ with accession number: PRJNA1110394.

## Author contributions

QL: Conceptualization, Data curation, Investigation, Software, Validation, Writing – original draft, Writing – review & editing. DT: Data curation, Investigation, Validation, Writing – original draft, Writing – review & editing. HC: Data curation, Investigation, Validation, Writing – original draft, Writing – review & editing. XG: Data curation, Software, Validation, Writing – original draft, Writing – review & editing. MA: Conceptualization, Methodology, Project administration, Writing – original draft, Writing – review & editing. XW: Data curation, Software, Supervision, Writing – review & editing. ZT: Conceptualization, Funding acquisition, Methodology, Project administration, Writing – original draft, Writing – review & editing. GP: Conceptualization, Funding acquisition, Investigation, Methodology, Project administration, Supervision, Writing – original draft, Writing – review & editing.
