# Supplementary material for: Endophyte-mediated enhancement of salt resistance in Arachis hypogaea L. by regulation of osmotic stress and plant defense-related genes
Source: Front Microbiol. 2024 May 23;15:1383545. doi: 10.3389/fmicb.2024.1383545 (PMC11153688; doi:10.3389/fmicb.2024.1383545)
Supplement: Supplementary file 1 [file Table_1.docx]

**Supplementary material Tables**

**Table S1** Endophytes were isolated from *M.lutarioriparius.*

| Number | type strain | similarity |
| --- | --- | --- |
| NDG1 | *Paenibacillus illinoisensis* NRRL NRS-1356 | 99.32% |
| NDG4 | *Flavobacterium acidificum* LMG 8364 | 97.76% |
| NDG5 | *Enterobacter chengduensis* WCHECl-C4 | 99.66% |
| NDG6 | *Pantoea* sp.YR343 | 99.41% |
| NDG7 | *Pseudomonas geniculata* ATCC 19374 | 99.01% |
| NDG8 | *Acinetobacter calcoaceticus* DSM 30006 | 100.0% |
| NDG12 | *Bacillus toyonensis* BCT-7112 | 99.59% |
| NDG14 | *Bacillus pacificus* EB422 | 99.9% |
| NDG15 | *Gottfriedia luciferensis* LMG 18422 | 99.67% |
| NDG19 | *Burkholderia gladioli* NBRC 13700 | 99.49% |
| NDG31 | *Stenotrophomonas maltophilia* MTCC 434 | 99.51% |
| NDG50 | *Bacillus ferrooxidans* YT-3 | 98.34% |
| NDG62 | *Priestia aryabhattai* B8W22 | 99.59% |
| NDG77 | *Ralstonia mannitolilytica* LMG 6866 | 98.93% |
| NDG81 | *Sinomonas flava* CW 108 | 99.27% |
| NDG82 | *Microbacterium binotii* CIP 101303 | 99.89% |
| NDJ1 | *Bacillus tequilensis* KCTC 13622 | 99.9% |
| NDJ20 | *Curtobacterium albidum* DSM 20512 | 98.69% |
| NDJ36 | *Neobacillus mesonae* FJAT-13985 | 99.9% |
| NDJ40 | *Staphylococcus epidermidis* NCTC 11047 | 99.8% |
| NDJ46 | *Brevibacillus borstelensis* NRRL NRS-818 | 100.0% |
| NDY2 | *Bacillus siamensis* KCTC 13613 | 99.89% |

**Table S2** Endophytes were tested for salt stress tolerance.

| Number | 0% NaCl | 2% NaCl | 4% NaCl | 8% NaCl | 12% NaCl | 16% NaCl |
| --- | --- | --- | --- | --- | --- | --- |
| NDG1 | + | + | + | - | - | - |
| NDG4 | + | + | + | + | - | - |
| NDG5 | + | + | + | - | - | - |
| NDG6 | + | + | + | - | - | - |
| NDG7 | + | + | + | - | - | - |
| NDG8 | + | - | - | - | - | - |
| NDG12 | + | + | + | - | - | - |
| NDG14 | + | + | + | - | - | - |
| NDG15 | + | - | - | - | - | - |
| NDG19 | + | - | - | - | - | - |
| NDG31 | - | - | - | - | - | - |
| NDG50 | + | + | - | - | - | - |
| NDG62 | + | + | + | + | + | + |
| NDG77 | + | - | - | - | - | - |
| NDG81 | + | + | + | - | - | - |
| NDG82 | + | + | - | - | - | - |
| NDJ1 | + | + | + | + | + | + |
| NDJ20 | + | + | - | - | - | - |
| NDJ36 | + | + | - | - | - | - |
| NDJ40 | + | + | + | + | + | + |
| NDJ46 | + | + | - | - | - | - |
| NDY2 | + | + | + | + | + | + |

Note: Sign “+” represents a function of resistance to salt stress, and “-” represents no.

**Table S3** A series of physiological and biochemical analyses of NDG62, NDJ1, NDJ40 and NDY2.

|  | NDG62 | NDJ1 | NDJ40 | NDY2 |
| --- | --- | --- | --- | --- |
| **Growth promotion test** |  |  |  |  |
| Protease | 1.85 ± 0.11 | 3.08 ± 0.20 | - | 2.98 ± 0.04 |
| CMC | - | 3.18 ± 0.42 | - | 3.34 ± 0.21 |
| Amylases | - | 1.78 ± 0.08 | - | 1.55 ± 0.09 |
| Iron carriers | - | + | + | + |
| P-solubilization | 1.13 ± 0.04 | - | 1.71 ± 0.54 | 1.35 ± 0.08 |
| **physiological and biochemical analyses** |  |  |  |  |
| VP test | - | + | + | + |
| Methyl red test | + | + | + | + |
| indole | - | + | - | + |
| urease | + | - | - | - |
| gelatin liquefaction | + | + | + | + |
| sucrose utilization | + | + | + | + |
| citrate utilization | + | + | + | + |
| ammonia production | + | - | - | - |

Note: The shown as “mean ± standard deviation” refers to the value of L/D, where “L” stands for the diameter of the circle of effect and “D” represents the diameter of the colony. The symbol “+” means with function, “-” means without.

**Table S4** Influence of “G62 + J1 + J40”, “G62 + J1 + Y2” and “G62 + J1 + J40 + Y2” on plant length, root length and biomass of peanut under different gradients of salt stress.

| **Treatment** |  | **Plant length**  **(cm)** |  | **Root length**  **(cm)** |  | **Plant biomass**  **(g FW)** |  | **Plant biomass**  **(g DW)** |
| --- | --- | --- | --- | --- | --- | --- | --- | --- |
| G62 + J1 + J40 |  |  |  |  |  |  |  |  |
| 0 mM L^-1^ NaCl |  | 4.71 ± 1.04 |  | 1.99 ± 0.91 |  | 2.26 ± 0.39 |  | 0.68 ± 0.07 |
| 50 mM L^-1^ NaCl |  | 3.62 ± 0.46 |  | 2.28 ± 0.60 |  | 2.43 ± 0.67 |  | 0.70 ± 0.09 |
| 100 mM L^-1^ NaCl |  | 1.55 ± 0.84 |  | 1.31 ± 0.83 |  | 2.18 ± 0.46 |  | 0.59 ± 0.09 |
| 200 mM L^-1^ NaCl |  | - |  | - |  | - |  | - |
| 250 mM L^-1^ NaCl |  | - |  | - |  | - |  | - |
| G62 + J1 + Y2 |  |  |  |  |  |  |  |  |
| 0 mM L^-1^ NaCl |  | 5.94 ± 0.69 |  | 4.78 ± 2.09 |  | 2.95 ± 0.08 |  | 1.12 ± 0.14 |
| 50 mM L^-1^ NaCl |  | 2.07 ± 0.25 |  | 1.46 ± 0.67 |  | 2.63 ± 0.81 |  | 0.82 ± 0.21 |
| 100 mM L^-1^ NaCl |  | - |  | - |  | - |  | - |
| 200 mM L^-1^ NaCl |  | - |  | - |  | - |  | - |
| 250 mM L^-1^ NaCl |  | - |  | - |  | - |  | - |
| G62 + J1 + J40 + Y2 |  |  |  |  |  |  |  |  |
| 0 mM L^-1^ NaCl |  | 1.83 ± 1.65 |  | 1.25 ± 1.50 |  | 2.83 ± 2.47 |  | 0.88 ± 0.76 |
| 50 mM L^-1^ NaCl |  | 2.86 ± 1.34 |  | 2.83 ± 0.56 |  | 3.44 ± 0.60 |  | 0.95 ± 0.14 |
| 100 mM L^-1^ NaCl |  | 2.08 ± 0.39 |  | 1.65 ± 0.70 |  | 2.83 ± 0.36 |  | 0.83 ± 0.16 |
| 200 mM L^-1^ NaCl |  | 1.06 ± 0.55 |  | 1.20 ± 0.56 |  | 2.78 ± 0.35 |  | 0.80 ± 0.06 |
| 250 mM L^-1^ NaCl |  | - |  | - |  | - |  | - |

Note: Difference in various growth parameters and comparative analysis. Average ± standard error from three separate replicates (n = 3). Different letters mean significantly different based on *p* < 0.05. “*”, “**”, and “***” indicate significant differences at *p* < 0.1, *p* < 0.05, and *p* < 0.01, respectively, and “ns” represents no significant difference.

**Table S5** Two-way ANOVA for different plant growth parameters under treatments and control.

| **Treatment** | **Stem lengtht**  **(cm)** | **Root length**  **(cm)** | **Root area**  **(cm^2^)** | **Leaf area**  **(cm^2^)** | **Stem biomass**  **(g FW)** | **Root biomass**  **(g FW)** | **Stem biomass**  **(g DW)** | **Root biomass**  **(g DW)** |
| --- | --- | --- | --- | --- | --- | --- | --- | --- |
| **Non-stress** |  |  |  |  |  |  |  |  |
| CK | 25.12 ± 2.61b | 21.50 ± 2.78b | 56.83 ± 11.50a | 89.06 ± 18.05b | 7.70 ± 1.85b | 2.86 ± 0.54a | 1.47 ± 0.30b | 0.22 ± 0.04b |
| TSB | 27.38 ± 1.20a | 24.63 ± 3.41a | 62.18 ± 11.99a | 151.51 ± 19.71a | 10.39 ± 1.70a | 3.30 ± 0.57a | 2.01 ± 0.31a | 0.29±0.05a |
| **Salt-stress** |  |  |  |  |  |  |  |  |
| NaCl | 17.34 ± 2.91d | 12.09 ± 1.90d | 19.86 ± 6.10b | 34.09 ± 9.22d | 3.99 ± 0.50c | 1.56 ± 0.44b | 0.99 ± 0.14c | 0.12 ± 0.02c |
| NaCl_TSB | 20.50 ± 1.76c | 16.15 ± 2.14c | 28.45 ± 5.74b | 59.85 ± 17.87c | 5.07 ± 1.59c | 1.80 ± 0.35b | 1.07 ± 0.17c | 0.14 ± 0.02c |
| **ANOVA** |  |  |  |  |  |  |  |  |
| TSB | ** | *** | * | *** | ** | * | ** | ** |
| Salt | *** | *** | *** | *** | *** | *** | *** | *** |
| TSB × Salt | ns | ns | ns | ** | ns | ns | ** | ns |

Note: “Non-stress” represents peanut grown without NaCl condition; “Salt stress” represents peanut under 200 mM L^-1^ salt conditions. Average ± standard error from nine separate replicates (n = 9). Different letters mean significantly different based on *p* < 0.05. “*”, “**”, and “***” indicate significant differences at *p* < 0.1, *p* < 0.05, and *p* < 0.01, respectively, and “ns” represents no significant difference.

**Table S6.** Two-way ANOVA for the effects of Non-Salt content LB (CK) and Bacteria (TSB), Salt concentration (NaCl), and Salt-stress inoculation with a bacterial strain (NaCl_TSB) on SOD, POD, CAT, PPO, MDA and Chlorophyll of peanut.

| Leaf | SOD (U g^−1^) | |  | POD(∆OD470 g^-1^  min^-1^) | |  | CAT(μM g^-1^ min^-1^ ) | |  | PPO (∆OD_420_ g^-1^ min^-1^) | |  | MDA (nM g^-1^ ) | |  | Chlorophyll(Spad) | |
| --- | --- | --- | --- | --- | --- | --- | --- | --- | --- | --- | --- | --- | --- | --- | --- | --- | --- |
| **Non-stress** |  |  |  |  |  |  |  |  |  |  |  |  |  |  |  |  |  |
| CK | 17.26 ± 1.90b | |  | 530.55 ± 168.05b | |  | 614.89 ± 17.72b | |  | 865.33 ± 161.56b | |  | 21.10 ± 1.67c | |  | 35.41 ± 2.01c | |
| TSB | 20.75 ± 2.65a | |  | 330.77 ± 132.40c | |  | 721.20 ± 4.68a | |  | 1569.33 ± 379.66a | |  | 20.52 ± 2.85c | |  | 37.93 ± 2.24bc | |
| **Salt-stress** |  | |  |  |  |  |  |  |  |  |  |  |  |  |  |  |  |
| NaCl | 11.57 ± 1.74c | |  | 736.77 ± 261.52a | |  | 465.35 ± 32.74c | |  | 1030.22 ± 325.43b | |  | 36.96 ± 3.10a | |  | 41.56 ± 5.92b | |
| NaCl_TSB | 14.81 ± 4.07b | |  | 628.00 ± 111.03ab | |  | 704.95 ± 6.23a | |  | 1579.11 ± 342.11a | |  | 31.72 ± 1.03b | |  | 46.45 ± 4.44a | |
| **ANOVA** | F | *p* |  | F | *p* |  | F | *p* |  | F | *p* |  | F | *p* |  | F | *p* |
| TSB | 13.41 | < 0.001 |  | 6.77 | 0.01 |  | 744.15 | < 0.001 |  | 35.92 | < 0.001 |  | 14.07 | < 0.001 |  | 30.27 | < 0.001 |
| Salt | 40.06 | < 0.001 |  | 18.03 | < 0.001 |  | 170.94 | < 0.001 |  | 0.69 | 0.41 |  | 304.79 | < 0.001 |  | 7.71 | < 0.01 |
| TSB × Salt | 0.02 | 0.88 |  | 0.58 | 0.44 |  | 110.48 | < 0.001 |  | 0.55 | 0.46 |  | 9.06 | 0.005 |  | 0.78 | 0.382 |

Note: The various letters indicate significant differences among different treatments at the *p* < 0.05 level, and data from all treatments were combined and analyzed jointly (n = 9). Average ± standard error from nine separate replicates. “F” indicates degrees of freedom, and “*p*” is a value for significant difference. “Non-stress” represents peanut grown without NaCl condition; “Salt stress” represents peanuts under 200 mM L^-1^ salt conditions.

**Table S7** Summary of sequencing data quality.

| **Sample** | **Raw Data** | **Raw bases/G** | **Clean Data** | **Clean bases/G** | **Ratio** | **Q20/%** | **Q30/%** | **GC/%** |
| --- | --- | --- | --- | --- | --- | --- | --- | --- |
| CK_1 | 54198140 | 8.13G | 52990196 | 7.95G | 0.98 | 98.00% | 94.17% | 43.99% |
| CK_2 | 55248228 | 8.29G | 54249946 | 8.14G | 0.98 | 97.90% | 93.85% | 44.06% |
| CK_3 | 39382780 | 5.91G | 38679332 | 5.80G | 0.98 | 98.16% | 94.52% | 44.08% |
| TSB_1 | 45030180 | 6.75G | 44164780 | 6.62G | 0.98 | 97.91% | 93.91% | 44.00% |
| TSB_2 | 54580164 | 8.19G | 53504598 | 8.03G | 0.98 | 98.01% | 94.30% | 44.20% |
| TSB_3 | 53557838 | 8.03G | 52474490 | 7.87G | 0.98 | 97.94% | 94.14% | 43.92% |
| NaCl_1 | 52397336 | 7.86G | 51279040 | 7.69G | 0.98 | 97.88% | 94.07% | 44.63% |
| NaCl_2 | 56666112 | 8.50G | 55454916 | 8.32G | 0.98 | 98.05% | 94.45% | 44.21% |
| NaCl_3 | 55111234 | 8.27G | 53944266 | 8.09G | 0.98 | 97.78% | 93.64% | 44.08% |
| NaCl_TSB_1 | 53682488 | 8.05G | 52536500 | 7.88G | 0.98 | 97.80% | 93.73% | 44.17% |
| NaCl_TSB_2 | 61957362 | 9.29G | 60500048 | 9.08G | 0.98 | 97.77% | 93.74% | 44.12% |
| NaCl_TSB_3 | 46185886 | 6.93G | 45128682 | 6.77G | 0.98 | 97.82% | 93.77% | 44.05% |

Note: Raw Data represent to Number of read pairs in the original data. Raw bases refers to total number of bases in the original data. Clean data refers to Count of read pairs in the cleared data. Clean bases refers to total number of bases in the clean data. Ratio refers to the ratio of data after clearance. Q20 refers to percentage of raw bases with Phred values greater than 20. Q30 refers to percentage of raw bases with Phred values greater than 30. GC refers to percentage of total G bases and C bases of all bases in raw reads.

**Table S8** Comparison and matching analysis of RNA-seq.

| **Sample** | **Total reads** | **Total Mapped reads** | **Total Mapped ratio(%)** | **Proper Mapped reads** | **Proper Mapped ratio(%)** |
| --- | --- | --- | --- | --- | --- |
| CK_1 | 59731492 | 57466067 | 96.21% | 47745228 | 93.79% |
| CK_2 | 61417772 | 59393881 | 96.70% | 49636260 | 94.34% |
| CK_3 | 44683833 | 43366555 | 97.05% | 35221006 | 94.85% |
| TSB_1 | 49567834 | 47367716 | 95.56% | 39717192 | 93.08% |
| TSB_2 | 60625467 | 58357867 | 96.26% | 48245440 | 93.90% |
| TSB_3 | 58943681 | 56337669 | 95.58% | 46849608 | 93.05% |
| NaCl_1 | 58240377 | 55647518 | 95.55% | 45457158 | 92.99% |
| NaCl_2 | 63916574 | 61354771 | 95.99% | 49907616 | 93.50% |
| NaCl_3 | 60423073 | 58515263 | 96.84% | 48355504 | 94.53% |
| NaCl_TSB_1 | 59480796 | 57049962 | 95.91% | 46811640 | 93.46% |
| NaCl_TSB_2 | 66169824 | 61348011 | 92.71% | 50539674 | 89.71% |
| NaCl_TSB_3 | 51096551 | 48947321 | 95.79% | 40474302 | 93.32% |

Note: Total reads means reads after quality control of sequencing data. Total Mapped reads refers to reads matched to the genome. Total Mapped ratio refers to percentage of reads matched to the genome. Proper Mapped reads means pairs of read1 and read2 were simultaneously compared to the number of reads in the genome. Percentage of paired read1 and read2 simultaneously compared to genomic reads. Proper Mapped ratio means percentage of Pairs of read1 and read2 are compared simultaneously.
